# Immunometabolism in Obesity-Associated Type 2 Diabetes: Molecular Mechanisms and Emerging Therapeutic Targets

**DOI:** 10.3390/ijms27188063

**Published:** 2026-09-10

**Authors:** Carlo Acierno, Massimiliano Cavallo, Damiano D’Ardes, Andrea Boccatonda, Davide Nilo, Alfredo Caturano

**Affiliations:** 1 Department of Health Sciences (DiSS), University of Basilicata, Via dell’Ateneo Lucano 10, 85100 Potenza, Italy; 2 Department of Infectious Diseases, Azienda Ospedaliera Regionale San Carlo, 85100 Potenza, Italy; 3 Internal Medicine Unit, Degli Infermi Hospital of Narni, USL Umbria 2, 05035 Narni, Italy; massimilianocavallotr@gmail.com; 4 Department of Medicine and Aging Sciences, University of G. d’Annunzio Chieti and Pescara, 66100 Chieti, Italy; damiano.dardes@unich.it; 5 Istituto di Ricovero e Cura a Carattere Scientifico (IRCCS) Azienda Ospedaliero-Universitaria di Bologna, 40138 Bologna, Italy; andrea.boccatonda2@unibo.it; 6 Department of Advanced Medical and Surgical Sciences, University of Campania Luigi Vanvitelli, 80138 Naples, Italy; nilodavide@gmail.com; 7 Department of Human Sciences and Promotion of the Quality of Life, San Raffaele Roma University, 00166 Rome, Italy; alfredo.caturano@uniroma5.it

**Keywords:** immunometabolism, type 2 diabetes, insulin resistance, metaflammation, NLRP3 inflammasome, adipose tissue macrophages, mitochondrial dysfunction, beta-cell dysfunction, gut microbiota, macrophage metabolic reprogramming

## Abstract

Type 2 diabetes (T2D) is increasingly understood as a chronic, low-grade inflammatory disease in which immune and metabolic signalling are bidirectionally coupled. Nutrient excess drives glucolipotoxic stress in adipose tissue, liver, skeletal muscle and pancreatic islets, engaging innate immune sensors. Responding immune cells then reconfigure their own intermediary metabolism, and the resulting metabolites—succinate, which stabilises hypoxia-inducible factor-1α, and the itaconate that opposes it—themselves specify inflammatory output. These signals converge on NOD-, LRR- and pyrin domain-containing protein 3 (NLRP3) inflammasome assembly, gasdermin D-mediated pyroptosis and interleukin-1β (IL-1β) release, which interrupt insulin signalling through inhibitory serine phosphorylation of insulin receptor substrate-1. Mitochondrial dysfunction, impaired mitophagy and cytosolic mitochondrial DNA sensing sustain the loop, while gut barrier failure supplies a parallel systemic input that converges on beta-cell dysfunction. This narrative review synthesises these mechanisms and examines how metformin, glucagon-like peptide-1 receptor agonists, sodium–glucose cotransporter 2 inhibitors and inflammasome-directed agents intersect with them. The chain described is that of obesity-associated T2D, and the heterogeneity that limits its generalisation across the recognised subgroups of the disease is addressed explicitly rather than assumed away. We give particular weight to a dissociation that constrains the field: sustained IL-1β neutralisation reduced cardiovascular events without preventing incident diabetes. Establishing why is, in our view, the central question for immunometabolic therapeutics in T2D.

## 1. Introduction

Type 2 diabetes (T2D) arises from the convergence of two processes long studied in isolation: dysregulated nutrient and energy metabolism, and chronic activation of the innate immune system. The field of immunometabolism formalises the observation that these processes are not parallel but bidirectionally coupled—metabolic signals reprogram immune cell function, and immune mediators in turn impair metabolic signalling in parenchymal cells [1,2]. This conceptual shift reframes T2D as a disease of immune-metabolic crosstalk rather than a simple defect of insulin action or insulin secretion [3,4].

The epidemiological backdrop is well established: the global rise in obesity has been accompanied by a parallel rise in T2D, with shared upstream drivers in caloric excess, sedentary behaviour, and adipose tissue expansion [5]. What links obesity to T2D mechanistically is chronic, low-grade, non-resolving inflammation—a state distinct from classical infectious or autoimmune inflammation, originally termed “metaflammation,” that is sustained by metabolic rather than pathogen-derived signals [6,7]. Unlike acute inflammatory responses, metaflammation does not resolve and instead becomes a chronic feature of metabolically stressed tissues, with magnitude and duration tracking the degree and persistence of nutrient excess [8].

This narrative review traces a single mechanistic chain across multiple organ systems. Nutrient excess and ectopic lipid accumulation trigger glucolipotoxic and danger-associated signalling, which activates innate immune sensors—principally in macrophages—leading to NOD-, LRR- and pyrin domain-containing protein 3 (NLRP3) inflammasome assembly, pyroptotic cell death, and cytokine release. Cytokine signalling and lipid-derived second messengers then interfere with proximal and distal nodes of insulin signalling in adipose tissue, liver, and skeletal muscle, producing systemic insulin resistance. Concurrently, islet-resident immune cells and cytokine exposure accelerate beta-cell dysfunction and apoptosis, converting a compensable insulin-resistant state into overt hyperglycaemia; sustained hyperglycaemia and inflammation, in turn, are associated with microvascular and macrovascular complications, plausibly closing a feedback loop that further amplifies inflammatory tone [9,10]. This chain describes the obesity-associated form of T2D, which accounts for the majority of cases and for nearly all of the mechanistic evidence available; the heterogeneity of the disease and the limits it places on any single sequential model are addressed directly in Section 11.

Several themes recur across this chain and structure the sections that follow. First, macrophages are central but heterogeneous effectors—their polarisation state, tissue residency, and metabolic programming determine whether their net effect on adjacent parenchymal cells is protective or pathogenic [11,12]. Second, mitochondrial function is both a target and a driver of inflammatory signalling, such that mitochondrial dysfunction in immune and metabolic cells reinforces rather than merely accompanies inflammatory activation [7]. Third, the gut, the adipose organ, the liver, skeletal muscle, and the endocrine pancreas are not independent compartments but communicate through circulating lipids, cytokines, microbial metabolites, and hepatokines/myokines/adipokines, such that immunometabolic dysfunction in one tissue propagates to others [13]. Fourth, several intracellular signalling nodes—AMP-activated protein kinase (AMPK), mechanistic target of rapamycin (mTOR), phosphoinositide 3-kinase/protein kinase B (PI3K/AKT), nuclear factor-κB (NF-κB), c-Jun N-terminal kinase (JNK), and the sirtuins—function as integrators that translate nutrient and energy status directly into inflammatory or anti-inflammatory transcriptional output, providing a unifying molecular grammar across tissues [14].

The clinical reach of this framework extends beyond classical glycaemic outcomes: the inflammatory and metabolic substrates described here are shared with the vascular complications of insulin resistance, so that macrovascular disease in T2D is better read as an expression of the same underlying immunometabolic process than as an independent comorbidity [10,15].

Finally, this convergence of mechanisms has direct translational relevance. Several glucose-lowering agents already in clinical use—metformin, glucagon-like peptide-1 (GLP-1) receptor agonists, and sodium–glucose cotransporter 2 (SGLT2) inhibitors—exert measurable anti-inflammatory and immunomodulatory effects that are continuous with the immunometabolic pathways discussed throughout this review, and inflammasome- and cytokine-directed agents are now being tested as primary therapeutic strategies rather than incidental benefits of glycaemic control [16,17]. Multi-omic and single-cell profiling is beginning to resolve the cellular and molecular heterogeneity underlying these processes, raising the prospect of immunometabolic endotyping being used as a basis for precision intervention in T2D [1,18].

## 2. Search Strategy and Evidence Selection

This article was designed as a narrative review, not as a systematic review or meta-analysis, with the aim of integrating mechanistic and translational evidence into a single interpretive framework linking innate immune activation to metabolic dysfunction in T2D. The literature was identified through iterative searches of PubMed/MEDLINE and Scopus, complemented by manual screening of the reference lists of key articles and of relevant reviews. Searching began in January 2026 and the last search was performed on 20 July 2026; no lower date limit was applied, so that the foundational studies that established the field remained retrievable alongside contemporary work. Search terms combined controlled vocabulary (Medical Subject Headings in PubMed/MEDLINE) with free-text keywords for the core concepts of this review—immunometabolism, type 2 diabetes, insulin resistance, metaflammation, NLRP3 inflammasome, adipose tissue macrophages, mitochondrial dysfunction, gut microbiota, and the therapeutic classes considered in Section 13—in successive Boolean combinations. The complete search strings executed in both databases are reproduced verbatim in Appendix A. The nine PubMed/MEDLINE blocks, executed with a filter restricting records to those entered in the database on or before the last search date, retrieved 71,171 records, reducing to 67,176 after de-duplication across blocks; these figures are exactly reproducible from the strings and dates given in Appendix A. Equivalent counts are not reported for Scopus, and the reason is worth stating rather than leaving to inference: Scopus offers no filter on date of indexing, so a count obtained now would describe the database as it stands today rather than as it stood at the last search date, and would not correspond to the search actually performed. We report the figure that can be reproduced and state why the other cannot. Consistent with a narrative rather than a systematic design, these records were not screened exhaustively. The results of each block were examined in order of relevance until further eligible material ceased to be identified, and the eligibility criteria and evidence hierarchy set out below were then applied to the records so retained. A formal record-level screening log was therefore not generated, and a count of records screened in the sense required by the Preferred Reporting Items for Systematic Reviews and Meta-Analyses (PRISMA) is not available. The final narrative synthesis includes 185 cited sources, comprising the complete reference list of this review. A small number of sources were identified after the last search date, in the course of peer review, and are included where they bear directly on a point raised; these are the only references in the list that postdate 20 July 2026.

**Figure 1:**
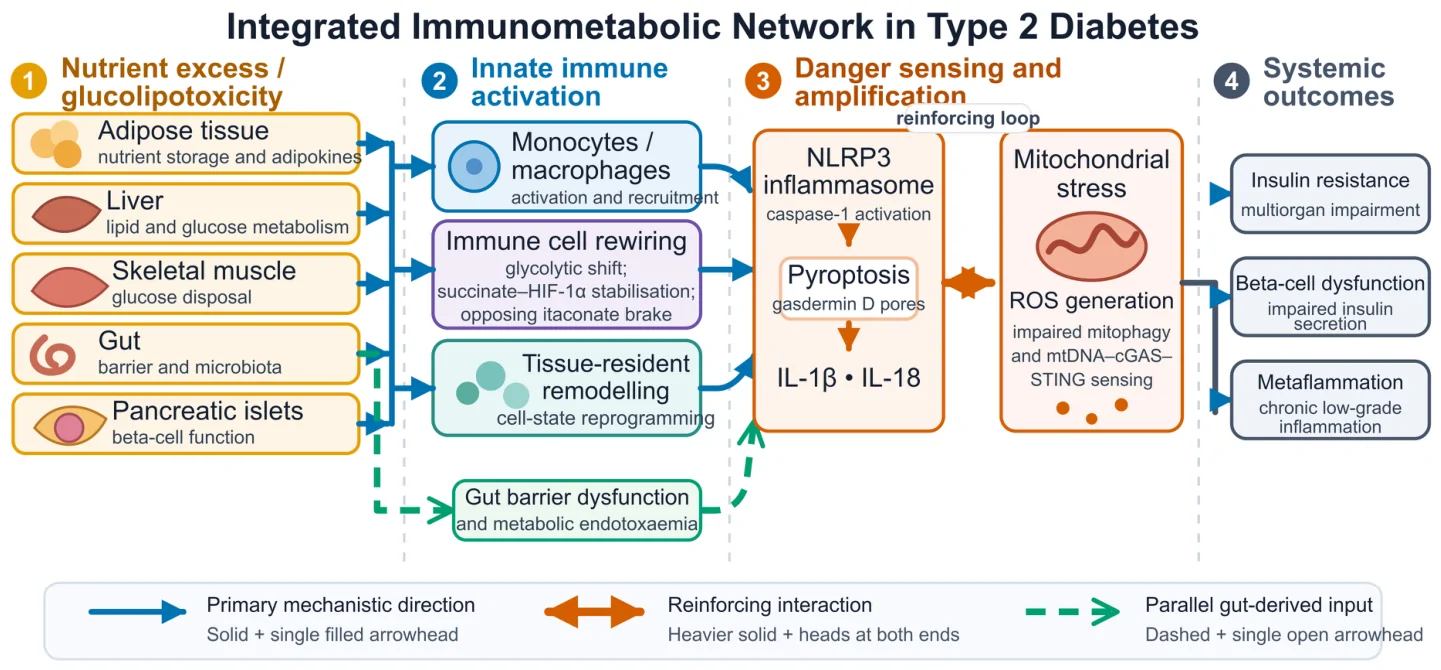
Integrated immunometabolic network in type 2 diabetes (T2D). The schematic is organised as a left-to-right mechanistic flow. Nutrient excess and glucolipotoxicity feed into five metabolic tissues—adipose tissue, liver, skeletal muscle, gut, and pancreatic islets—which drive innate immune activation. Three processes are distinguished within that activation: recruitment and activation of monocytes/macrophages; reconfiguration of the immune cell’s own intermediary metabolism (glycolytic shift, succinate accumulation with hypoxia-inducible factor-1α (HIF-1α) stabilisation, and the opposing itaconate brake); and tissue-resident immune remodelling. These converge on a danger-sensing and amplification module comprising the NOD-, LRR- and pyrin domain-containing protein 3 (NLRP3) inflammasome, gasdermin D-mediated pyroptosis, and interleukin-1β (IL-1β)/interleukin-18 (IL-18) signalling, coupled with mitochondrial stress, reactive oxygen species (ROS) generation, impaired mitophagy, and cytosolic mitochondrial DNA (mtDNA) sensing through cyclic GMP-AMP synthase (cGAS) and stimulator of interferon genes (STING). Arrow semantics are encoded redundantly by colour, line style and arrowhead, so that the figure remains interpretable in greyscale and to readers with colour vision deficiency: solid single-headed arrows show the primary direction of mechanistic influence; the solid, heavier, double-headed arrow denotes the reinforcing interaction between inflammasome/cytokine signalling and mitochondrial stress; and the dashed arrow denotes the parallel gut-derived input through barrier dysfunction and metabolic endotoxaemia. The palette follows the Okabe–Ito colour-vision-deficiency-safe scheme, and the figure has been checked under simulated deuteranopia and protanopia and in greyscale. Arrow colour additionally identifies the module of origin and carries no independent meaning. The circled numerals 1–4 identify the four sequential stages of the network and carry no further meaning; the orange dots within the mitochondrial stress box denote, schematically, reactive oxygen species and released mitochondrial DNA; and the raised dot between IL-1β and IL-18 is a separator between the two cytokines and does not denote an operation. The downstream outputs are insulin resistance, beta-cell dysfunction, and chronic low-grade inflammation (metaflammation). The flow depicted is the dominant sequence in obesity-associated T2D and should be read as the prevailing direction of influence rather than as an obligatory order: as discussed in Section 11, insulin-deficient and lean phenotypes may enter the network at the beta-cell node with comparatively little upstream adipose input. The immune cell rewiring node—glycolytic shift, succinate–HIF-1α stabilisation and the opposing itaconate brake—is drawn from experimental work in acutely stimulated murine macrophages and remains hypothetical in human T2D; this evidence tier is stated in the corresponding summary table and discussed in Section 5 and Section 15. The figure was prepared by the authors using Microsoft PowerPoint (Microsoft Corporation, Redmond, WA, USA) and Inkscape v1.3 (Inkscape Project, free and open-source vector graphics software; https://inkscape.org, accessed on 20 July 2026).

Records were considered eligible if they were published in English in a peer-reviewed journal and addressed at least one of three domains. The first was a molecular or cellular mechanism linking innate immune activation to insulin resistance or to beta-cell dysfunction. The second was a clinical study reporting metabolic, inflammatory or cardiovascular endpoints for an agent acting on one of those mechanisms. The third was a conceptual framework bearing on the interpretation of either. Conference abstracts, preprints, case reports, editorials without primary content, and studies reporting associations without a mechanistic or clinical endpoint were not used to support substantive claims. Where several publications addressed the same mechanism, preference was given to the most recent and mechanistically informative source, and to human over animal data of equivalent quality. No formal PRISMA-based search strategy, risk-of-bias assessment, or quantitative evidence synthesis was performed. Evidence was instead prioritised according to an explicit interpretive hierarchy: consensus statements and clinical guidelines first, followed by randomised controlled trials and meta-analyses, mechanistic studies conducted in human tissue, and, where human data were unavailable, experimental and animal studies used to support biological plausibility and explicitly identified as such. Findings resting on a single primary report are identified as such at the point of citation. The tier of the underlying evidence is stated explicitly for every mechanism summarised in the summary tables of this review and for the immune cell rewiring node of Figure 1, so that the hierarchy set out here can be applied by the reader rather than merely asserted by us. Selection and weighting were performed by the first author and reviewed by the co-authors, with disagreements resolved by discussion against the same hierarchy.

Because the strength of the underlying evidence varies substantially across the mechanisms discussed, claims of clinical efficacy are qualified throughout by the corresponding endpoint, and mechanistic plausibility is consistently distinguished from demonstrated clinical benefit. This narrative approach permits the integration of heterogeneous evidence into a coherent mechanistic account but does not provide the reproducibility or formal bias control of a systematic review; that constraint, together with the restriction of searching to two bibliographic databases, is considered further in Section 15.

## 3. Nutrient Excess, Glucolipotoxicity and Metaflammation

Sustained positive energy balance is the proximate trigger of the immunometabolic cascade in the obesity-associated form of T2D with which this account is concerned (Section 11). When nutrient supply exceeds the storage and oxidative capacity of adipose tissue, lipids accumulate ectopically in liver, skeletal muscle, and the endocrine pancreas, and circulating free fatty acid and glucose concentrations rise chronically—the combined state of glucolipotoxicity [19]. Glucolipotoxic stress is not simply a quantitative excess of substrate; it generates qualitatively distinct intracellular signals—diacylglycerols, ceramides, and reactive lipid intermediates—that directly engage inflammatory kinases and pattern-recognition pathways in parenchymal and immune cells alike [20].

Which of these lipid species is the proximate mediator remains genuinely contested, and the disagreement is worth stating plainly because the two models imply different therapeutic targets. In the diacylglycerol model, lipid accumulation activates novel protein kinase C isoforms—PKCε in liver and PKCθ in skeletal muscle. These phosphorylate the insulin receptor and insulin receptor substrate (IRS) proteins on inhibitory residues, interrupting signal transmission at its most proximal node. Two lines of evidence support the model: the tight correlation between cytosolic diacylglycerol content and insulin resistance in human liver and muscle, and the protection conferred by isoform-specific knockdown [21,22]. In the ceramide model, sphingolipid intermediates act instead through protein phosphatase 2A and atypical PKCζ, blocking Akt activation downstream of the receptor. The strongest support comes from genetic and pharmacological manipulation of ceramide synthesis. More recently, desaturation of a single double bond in the ceramide backbone was shown to be required for its metabolic toxicity, and inhibiting the responsible desaturase improves insulin sensitivity and hepatic steatosis [23]. The two models are not mutually exclusive—they may operate in different tissues, at different stages, or at different degrees of lipid overload—but the field has not converged, and reviews that present lipotoxicity as a single unified mechanism obscure a distinction with direct consequences for drug development. We note it here rather than resolving it, since the immunometabolic arguments that follow are compatible with either.

Adipose tissue is the primary site at which nutrient excess is translated into an immunological signal. As adipocytes hypertrophy beyond their vascular and oxidative capacity, they become hypoxic and undergo a stress response characterised by abnormal adipokine secretion, increased lipolysis, and the release of damage-associated molecular patterns that recruit and activate resident and infiltrating immune cells [24,25]. This expanding, dysfunctional adipose depot secretes a shifted profile of adipokines, hepatokines, and myokines that act as long-range immunometabolic messengers, altering inflammatory tone in liver and skeletal muscle even before substantial ectopic lipid deposition occurs in those tissues [26].

The concept of metaflammation captures the resulting state: a chronic, low-intensity, tissue-distributed inflammatory response driven by metabolic, not infectious, signals, continuous with but distinguishable from classical inflammation [27,28]. Its empirical origin is a single observation: adipose tissue of obese rodents overexpresses tumour necrosis factor-α, and neutralising it improves insulin sensitivity—the first demonstration that a cytokine produced by fat tissue impairs insulin action, and the point at which obesity ceased to be understood as a purely metabolic state [29]. Unlike acute inflammatory responses that resolve once the inciting stimulus is cleared, metaflammation persists as long as nutrient excess and adipose tissue dysfunction persist, and progressively recruits additional tissues into the inflammatory network. A key feature distinguishing metaflammation from infection-driven inflammation is its initiation in the apparent absence of an overt pathogen, a feature that historically delayed recognition of inflammation as a core pathogenic mechanism in metabolic disease, and that is now explained by the capacity of nutrient excess itself to activate canonical innate immune sensors such as the NLRP3 inflammasome [30].

Mitochondrial stress in adipocytes is an early and possibly initiating event in this cascade, not a late consequence of inflammation. A mitochondrial–stress adipocyte–macrophage circuit has been described in human T2D adipose tissue in which adipocyte mitochondrial dysfunction directly signals to resident macrophages, sustaining a self-reinforcing metaflammatory loop independent of further nutrient input; this circuit has so far been reported in a single recent study and awaits independent replication [31]. Mitochondrial quality-control failure in adipose tissue carries systemic rather than merely local consequences for insulin sensitivity, a point developed in Section 8 [32].

Skeletal muscle is both a target and a contributor to this network. Obesity-associated skeletal muscle inflammation, driven in part by circulating lipid and cytokine signals from adipose tissue, impairs muscle glucose uptake and contributes to whole-body insulin resistance independent of direct lipid accumulation within myocytes [33]. The molecular machinery that couples nutrient sensing to inflammatory output at the cell-intrinsic level converges substantially on the PI3K/Akt axis, which is engaged both by insulin signalling and by inflammatory cytokine receptors, providing a structural basis for cross-talk between metabolic and inflammatory signalling at the single-cell level [20].

The gut represents an additional, often underappreciated, source of metaflammatory signal: diet-induced alterations in gut microbial composition and barrier function generate microbial ligands that reach the systemic circulation and engage innate immune receptors in adipose tissue and liver, a parallel input developed in full in Section 10 [34]. Adipose tissue dysfunction itself spans a developmental continuum, with paediatric-onset adipose tissue dysfunction predisposing to an earlier and more entrenched metaflammatory phenotype that persists into adulthood [24].

Finally, the GPSM1–macrophage axis illustrates how a single intracellular signalling node can determine the macrophage inflammatory set-point in response to nutrient excess: GPSM1 controls a pro-inflammatory signalling pathway in macrophages that is necessary for full expression of obesity-associated metabolic dysfunction, identifying it as a candidate node linking nutrient sensing to macrophage activation state [35]. The liver is a particularly informative site at which to observe this convergence. Hepatic steatosis and T2D have been explicitly characterised as sharing overlapping pathophysiological mechanisms: ectopic lipid accumulation, lipotoxic signalling and secondary inflammatory activation. Nonalcoholic fatty liver disease can therefore be regarded as the hepatic face of the same glucolipotoxic process that produces systemic insulin resistance, and not as a separate comorbidity [36]. Together, these observations establish nutrient excess and glucolipotoxicity not as background risk factors but as the initiating immunological event of the entire pathogenic cascade addressed in this review.

## 4. Innate Immune Activation in Insulin Resistance

The innate immune system is the principal effector arm translating metaflammatory signals into impaired insulin action in this setting. Macrophages are quantitatively and mechanistically dominant: their accumulation, activation state, and metabolic reprogramming in insulin-sensitive tissues correlate closely with the degree of systemic insulin resistance [37,38]. Under lean, metabolically healthy conditions, tissue-resident macrophages and other innate immune populations maintain a regulatory, anti-inflammatory phenotype that supports normal tissue remodelling and insulin sensitivity [39]. With nutrient excess, this homeostatic population is progressively outnumbered and functionally overridden by infiltrating, pro-inflammatory monocyte-derived macrophages, a transition that is both a marker and a mechanistic driver of insulin resistance [40,41].

Mechanistically, macrophage-derived cytokines—principally tumour necrosis factor-α (TNF-α), interleukin-6 (IL-6), and IL-1β—engage receptors on adipocytes, hepatocytes, and myocytes that activate serine/threonine kinases (notably JNK and IKKβ) capable of inhibitory serine phosphorylation of insulin receptor substrate proteins, thereby directly uncoupling the insulin receptor from downstream PI3K/Akt signalling [42]. This signalling pathway provides a direct molecular link between innate immune activation and insulin resistance that operates in parallel across multiple insulin-target tissues, explaining why inflammatory activation in adipose tissue alone is sufficient to produce hepatic and skeletal muscle insulin resistance through circulating mediators [43].

Beyond classical macrophages, multiple additional innate immune populations contribute to this network. Innate lymphoid cells, particularly group 1 subsets, expand in obese adipose tissue and skew the local cytokine milieu toward an insulin resistance-promoting profile, contrasting with the protective role of type 2 innate lymphoid cells under lean conditions [44]. Regulatory T cells resident in lean adipose tissue actively restrain inflammation and preserve insulin sensitivity; their depletion is sufficient to provoke age-associated insulin resistance even without additional metabolic insult, indicating that loss of immune regulatory tone—not only gain of pro-inflammatory signalling—drives the transition to insulin resistance [45].

The adaptive compartment is not a passive downstream reader of this innate activation, and treating it as such would misrepresent the sequence of events. CD8+ effector T cells have been reported to infiltrate adipose tissue before macrophages accumulate and to be required for their recruitment: depletion of CD8+ cells lowers macrophage content and improves insulin resistance in obese mice, whereas adoptive transfer aggravates both. This has been proposed to place a T cell response upstream of the macrophage biology that dominates most accounts of this field [46]. The temporal priority rests on a single murine model, has not been demonstrated in human adipose tissue, and is contested; the finding is more safely read as evidence that the two compartments are interdependent than as evidence of a fixed order. B cells contribute through a distinct mechanism, promoting insulin resistance by modulating T cell function and producing pathogenic IgG antibodies, such that B cell depletion is protective and the transfer of IgG from obese animals is sufficient to transmit the phenotype [47]. On the opposing side, type 2 innate lymphoid cells sustain the eosinophils and alternatively activated macrophages that maintain lean adipose tissue homeostasis, and their loss removes a tonic restraint on inflammation rather than adding a stimulus [48]. Adipose tissue immunity is therefore better described as a shifting balance between opposing lymphoid programmes than as a unidirectional progression from innate activation to tissue damage.

A particularly important recent development is the recognition that innate immune cells acquire durable, epigenetically encoded changes in response to metabolic stress—a phenomenon termed trained immunity or innate immune memory—that sustain heightened inflammatory responsiveness even after the initiating stimulus is removed [49,50]. Insulin signalling itself appears to facilitate this training process in macrophages through metabolic reprogramming, suggesting a feed-forward loop in which insulin-resistant, hyperinsulinaemic states paradoxically promote sustained macrophage activation [50]. This concept of metabolic innate immune memory, captured under the heading “train to lose” in metaflammation, implies that transient metabolic insults (e.g., a period of weight gain or a high-fat dietary exposure) can leave a persistent pro-inflammatory imprint on innate immune cells that outlasts the original metabolic perturbation [51]. Weight-cycling studies in adipose tissue macrophages have directly demonstrated that prior obesity leaves an innate immune memory that intensifies the inflammatory and metabolic response to subsequent weight regain, providing a plausible mechanism for the poor durability of weight-loss interventions on insulin sensitivity [52].

Trained immunity is not restricted to a single mechanism. Maladaptive trained immunity in adipose tissue macrophages has been proposed as an explicit unifying link between obesity, T2D, and downstream cardiovascular disease, in which epigenetically reprogrammed macrophages drive both local adipose inflammation and circulating mediators of vascular risk [53]. Trained immunity-like reprogramming has further been implicated in autoimmune contexts, illustrating that this mechanism is not specific to metabolic disease but represents a general principle of innate immune cell plasticity with particular relevance to chronic, non-resolving inflammatory states such as T2D [54].

Environmental and systemic exposures interact with this innate immune axis, although their contribution remains peripheral to the core mechanism. Micro- and nanoplastic exposure and chronic low-grade infectious states such as periodontitis have each been linked experimentally to altered macrophage polarisation and systemic innate immune activation. That evidence is largely preclinical and has not been quantified at population level in T2D [55,56]. At the systemic level, the early inflammatory trajectory of T2D—beginning before frank hyperglycaemia and progressing through distinct stages of innate immune engagement—has been proposed as a framework for timing immunomodulatory interventions earlier in disease evolution, before insulin resistance becomes entrenched [57].

Finally, innate immune activation in insulin resistance does not occur in isolation from cardiovascular and other tissue beds: macrophage-driven inflammation in insulin resistance shares its principal effector mechanisms with atherosclerosis, and the chronic inflammatory state of insulin resistance independently predisposes to cardiovascular and endocrine comorbidity through shared innate immune mechanisms [10,15]. The convergence of these innate immune mechanisms—macrophage polarisation, trained immunity, regulatory T cell loss, and innate lymphoid cell imbalance—raises a question that the preceding account cannot answer on its own: what determines, at the level of the individual immune cell, whether it adopts an inflammatory or a regulatory programme? The answer lies in the cell’s own intermediary metabolism, which is addressed next.

## 5. Metabolic Reprogramming of Immune Cells

Section 3 and Section 4 described immune cells acting upon metabolic tissue. The reverse relationship—how intermediary metabolism within the immune cell dictates its inflammatory output—constitutes immunometabolism in its narrower sense, and supplies the mechanistic layer that connects nutrient availability to effector function [58]. Metabolic intermediates in this framework are not merely fuel: several act as signalling molecules in their own right, directly modifying transcription factors, chromatin, and enzymes. This distinction matters for T2D, because it implies that the substrate environment of an inflamed tissue can specify immune cell behaviour independently of receptor-level stimulation.

The best-characterised example is the metabolic configuration adopted by inflammatory macrophages. Upon activation through Toll-like receptor 4 (TLR4)—the receptor engaged by both saturated fatty acids and microbiota-derived lipopolysaccharide (LPS)—macrophages shift from oxidative phosphorylation towards aerobic glycolysis, a Warburg-like reorientation that sacrifices ATP yield for biosynthetic flexibility and rapid effector output [58]. Alternatively activated macrophages, by contrast, sustain intact oxidative phosphorylation and fatty acid oxidation, a configuration that supports the tissue-remodelling and regulatory phenotype characteristic of lean adipose tissue. The polarisation states described earlier therefore correspond to distinct bioenergetic configurations, not merely to distinct cytokine repertoires.

This reorientation is accompanied by two interruptions in the tricarboxylic acid cycle, which convert it from a catabolic circuit into a generator of signalling metabolites. Succinate accumulates behind the block at succinate dehydrogenase and stabilises hypoxia-inducible factor-1α by inhibiting prolyl hydroxylases, which in turn sustains transcription of pro-interleukin-1β—establishing a direct, chemically defined route from a Krebs cycle intermediate to a cytokine central to insulin resistance [59]. Succinate dehydrogenase contributes further through reverse electron transport at complex I, repurposing mitochondria from ATP synthesis towards ROS production and thereby amplifying the inflammatory programme; pharmacological inhibition of succinate dehydrogenase attenuates it in murine macrophages [60]. This mechanism is of particular interest here because it makes mitochondrial dysfunction a determinant of immune cell phenotype rather than only a consequence of inflammation.

The second interruption, at isocitrate dehydrogenase, diverts citrate towards itaconate through the enzyme aconitate decarboxylase 1 (ACOD1/IRG1), and itaconate has emerged as an endogenous brake on precisely the programme just described. Itaconate is itself a succinate dehydrogenase inhibitor, limiting succinate oxidation and restraining the inflammatory output of activated macrophages [61]. It acts additionally through covalent modification: by alkylating cysteine residues on Kelch-like ECH-associated protein 1 (KEAP1), itaconate releases nuclear factor erythroid 2-related factor 2 (NRF2) to drive an antioxidant transcriptional programme, and the cell-permeable derivative 4-octyl itaconate reproduces this anti-inflammatory effect in murine models [62]. An endogenous metabolite that simultaneously restrains succinate signalling and activates antioxidant defence is an attractive candidate node in a disease defined by the convergence of both, although itaconate-directed compounds have not yet been tested in human metabolic disease. Amino acid handling supplies a further, less classical input into the same programme: excitatory amino acid transporters have been shown to support the inflammatory responses of activated macrophages, indicating that the substrate dependencies of the inflammatory phenotype extend beyond glucose and the tricarboxylic acid cycle intermediates considered so far [63].

Two qualifications should temper the transfer of this framework to human T2D. First, most of the evidence derives from bone marrow-derived macrophages given acute, high-dose TLR stimulation, whereas adipose tissue macrophages in T2D experience chronic, low-intensity lipid and glucose stress; whether the same discrete metabolic switches operate under those conditions, or whether they represent graded and partially reversible states, is not established. Second, the immunometabolic literature has been built predominantly in murine systems, and human adipose tissue macrophages differ appreciably in surface phenotype and lipid handling. Human data addressing this question directly remain limited, and single-cell approaches (Section 14) currently offer the most realistic route to resolving it.

Even with those caveats, this layer supplies something the preceding sections lacked: a mechanism by which the nutrient excess described earlier could specify immune cell behaviour directly, without an intervening receptor-mediated signal. It also identifies the immediate substrate of the next section, since succinate accumulation, mitochondrial ROS generated by reverse electron transport, and the metabolites that oppose them all converge on the assembly of the NLRP3 inflammasome.

## 6. NLRP3 Inflammasome, Pyroptosis and Cytokine Signalling

The NLRP3 inflammasome is the central molecular platform through which diverse metabolic danger signals are converted into mature, bioactive IL-1β and IL-18, and it occupies a privileged position in the immunometabolic cascade as the principal proximate driver of cytokine-mediated insulin resistance in obesity-associated disease [30,64]. NLRP3 inflammasome activation in T2D follows the canonical two-signal model: a priming signal—typically saturated fatty acids, hyperglycaemia, or TLR ligands acting through NF-κB—upregulates NLRP3 and pro-IL-1β expression, while a second signal, often involving mitochondrial ROS, lysosomal destabilisation, or potassium efflux, triggers inflammasome assembly and caspase-1 activation [64]. NLRP3 itself is a tripartite sensor—an N-terminal pyrin domain, a central nucleotide-binding NACHT domain, and C-terminal leucine-rich repeats—and on activation it oligomerises through NACHT-domain interactions and recruits, via homotypic pyrin-domain binding, the adaptor apoptosis-associated speck-like protein containing a caspase activation and recruitment domain (ASC), whose CARD then nucleates pro-caspase-1 into the active protease [30]. Saturated fatty acids are a specific and directly relevant second signal: palmitate activates the NLRP3-ASC inflammasome in macrophages at 200 to 500 μM, applied for 24 h to bone marrow-derived macrophages primed with lipopolysaccharide, and thereby interferes with insulin signalling in adjacent cells, whereas the unsaturated fatty acids discussed below do not [65]. That concentration is of the order of plasma palmitate levels in obesity, but the priming and exposure conditions are acute and do not reproduce the chronic, low-intensity lipid stress of adipose tissue in T2D—a qualification that applies to much of the macrophage literature drawn on in Section 5. Thioredoxin-interacting protein (TXNIP) has been identified as a key molecular link between oxidative stress and NLRP3 activation, dissociating from oxidised thioredoxin under high ROS conditions to bind and activate NLRP3 directly, thereby coupling mitochondrial oxidative stress mechanistically to inflammasome-driven inflammation [64].

Once assembled, the inflammasome triggers caspase-1-dependent cleavage of gasdermin D at Asp275, which separates the pore-forming N-terminal domain from its autoinhibitory C-terminal partner; the liberated fragment binds acidic membrane phospholipids and oligomerises into transmembrane pores, driving the lytic, pro-inflammatory form of cell death known as pyroptosis [66]. The same cleavage is executed by caspase-11 (caspase-4/5 in humans) in response to cytosolic LPS, providing a route from gut-derived endotoxin to pyroptotic cell death that bypasses the canonical inflammasome entirely [67]. Pyroptotic release of IL-1β, IL-18, and additional intracellular danger signals amplifies local inflammation and recruits further innate immune cells, creating a self-sustaining inflammatory focus within metabolically stressed tissue [68]. Nucleotide metabolic rewiring within obese adipose tissue has recently been shown to enable hyperactivation of the NLRP3 inflammasome beyond the levels achieved by lipid signals alone, indicating that intracellular metabolite pools—not only extracellular lipid and glucose excess—actively license inflammasome hyperactivation in obesity; this mechanism rests at present on a single primary report and awaits independent replication [69].

The downstream consequences of IL-1β signalling for insulin action are direct and well characterised: IL-1β receptor engagement activates IRAK/NF-κB and JNK signalling in adipocytes and hepatocytes, producing inhibitory serine phosphorylation of insulin receptor substrate-1 and thereby attenuating PI3K/Akt-dependent glucose uptake, an effect that is reversible upon IL-1β pathway blockade in experimental models [30]. This mechanistic link has been tested directly in humans, and the results define both the promise and the limits of the framework advanced in this review. In a 13-week randomised trial in 70 patients with T2D, the IL-1 receptor antagonist anakinra lowered glycated haemoglobin by 0.46 percentage points relative to placebo, enhanced C-peptide secretion, and reduced circulating inflammatory markers—establishing that IL-1 signalling is not merely correlated with impaired beta-cell function but contributes causally to it [70]. The larger and more instructive test came from CANTOS, in which 10,061 post-infarction patients with high-sensitivity C-reactive protein ≥ 2 mg/L were randomised to placebo (*n* = 3344) or to the IL-1β-neutralising antibody canakinumab at 50 mg (*n* = 2170), 150 mg (*n* = 2284) or 300 mg (*n* = 2263) subcutaneously every three months; the trial met its cardiovascular endpoint and confirmed that interrupting this cytokine axis alters clinical outcomes [71]. Its pre-specified metabolic analysis, however, was negative. Of those randomised, 4960 (49.3%) met the protocol definition of pre-diabetes at trial entry. Over a median follow-up of 3.7 years, the incidence of adjudicated new-onset type 2 diabetes was 4.2, 4.2, 4.4 and 4.1 per 100 person-years in the placebo, 50 mg, 150 mg and 300 mg groups, respectively (log-rank *p* = 0.84), giving a hazard ratio of 1.02 (95% confidence interval [CI] 0.87–1.19; *p* = 0.82) for all canakinumab doses combined versus placebo. A reduction in glycated haemoglobin over the first 6 to 9 months of treatment was not sustained, and no consistent long-term effect on glycated haemoglobin or fasting plasma glucose was observed [72].

The two trials are often read as a single interleukin-1 narrative that ends in a paradox: blocking the pathway improved glycaemia in one setting and prevented nothing in the other. That reading is available only if the two studies are treated as interchangeable tests of one hypothesis. They are not. They differ simultaneously along three axes—the molecular selectivity of the agent, the population and stage of disease, and the endpoint through which effect was ascertained—and each axis is on its own sufficient to dissociate the results. Table 1 sets the two studies side by side on these axes.

The first axis is the selectivity of the agent. Anakinra is recombinant interleukin-1 receptor antagonist and acts as a competitive antagonist at IL-1R1, so that it blocks signalling by both IL-1α and IL-1β [70]. Canakinumab is a monoclonal antibody that neutralises circulating IL-1β alone [71]. The distinction is not academic in metabolic tissue: IL-1α is constitutively present in an active precursor form, is released as an alarmin from stressed and dying cells, and can signal in a membrane-associated form over short distances without proteolytic processing [73]. A hypertrophic, hypoxic and partly necrotic adipose depot is precisely the setting in which such a locally acting ligand would be expected to matter. Neutralising IL-1β while leaving IL-1α able to engage the same receptor therefore does not reproduce the pharmacology of receptor-level blockade, and residual IL-1α signalling remains a candidate explanation for the preserved inflammatory tone at the tissue level.

The second axis is the population and the stage of disease. The anakinra trial enrolled 70 patients with established T2D, in whom islet inflammatory stress is active and the target is engaged at the site where the drug is expected to act [70]. The CANTOS metabolic analysis asked a prevention question in a different population: post-infarction patients selected for high-sensitivity C-reactive protein ≥ 2 mg/L, that is, selected for vascular rather than for metabolic inflammation, of whom 4960 (49.3%) met the protocol definition of pre-diabetes [72]. Entry criteria that enrich for atherosclerotic inflammation do not necessarily enrich for the adipose–islet process described in this review, and in participants with pre-diabetes the beta-cell trajectory has typically been established over years before randomisation.

The third axis is the endpoint, and which arm of the mechanism it interrogates. This is the most informative of the three. Anakinra improved glycated haemoglobin and beta-cell secretory function—C-peptide secretion was enhanced and the proinsulin-to-insulin ratio fell—while insulin resistance measured by hyperinsulinaemic–euglycaemic clamp, insulin-regulated gene expression in skeletal muscle, serum adipokine levels and body-mass index were all similar between the two groups [70]. Interleukin-1 blockade in established disease therefore acted on secretion and not on sensitivity. Progression from pre-diabetes to diabetes, by contrast, is driven predominantly by worsening insulin resistance and weight gain, and is ascertained as a dichotomous adjudicated event defined by a diagnostic threshold. It follows that even if canakinumab had reproduced the beta-cell effect of anakinra in full, a reduction in incident diabetes would not necessarily have been observed. The arm of the mechanism on which IL-1 blockade demonstrably acts is not the arm that predominantly determines conversion, and a continuous improvement of the order of 0.46 percentage points of glycated haemoglobin need not move individuals across a diagnostic threshold.

Two further observations constrain the interpretation. First, target engagement in CANTOS was not in doubt: the incidence of new-onset diabetes was unchanged despite large reductions in both high-sensitivity C-reactive protein and interleukin-6 [72]. The negative result therefore concerns the consequences of IL-1β neutralisation, not whether the pathway was blocked. Second, the same analysis confirmed that baseline high-sensitivity C-reactive protein was itself prognostic of incident diabetes, with incidence rates rising across tertiles from 3.2 to 4.1 to 4.4 per 100 person-years (*p* = 0.003) [72]. Inflammation thus marks the risk without IL-1β neutralisation modifying it, which is the sharpest available statement of the dissociation and one that argues against reading the CANTOS result as evidence that inflammation is epiphenomenal.

Read together, and not against one another, the two trials delimit where interleukin-1 blockade has been shown to act and where it has not: in established disease, on beta-cell secretory function, ascertained by continuous glycaemic and secretory measures, with an agent that blocks the receptor rather than a single ligand. They generate a corresponding and testable prediction—that the informative trial has not yet been done, and would combine receptor-level or dual-ligand blockade, a population with established or early-established disease, and beta-cell functional rather than incident-diagnosis endpoints.

What remains genuinely unresolved is why residual signalling suffices. Three readings remain compatible with the evidence and are not mutually exclusive. IL-1β may be one of several partially redundant effectors, so that neutralising it leaves the network largely intact. The relevant compartment may be the islet and adipose microenvironment and not the circulation, so that systemic neutralisation fails to achieve adequate local exposure. Or the durable component of the response may be confined to a subset of patients. The 39-week follow-up of the anakinra trial bears on the last of these: the improvement in the proinsulin-to-insulin ratio and in inflammatory markers persisted after withdrawal, whereas the gain in stimulated C-peptide did not, except in a subgroup characterised by genetically determined low baseline IL-1Ra levels [74]. That observation connects this question directly to the heterogeneity considered in Section 11: the relevant unit of analysis may not be the disease but the patient subgroup, and no trial reported to date has been designed on that basis.

Inflammasome activation in T2D also intersects with cellular senescence and tissue ageing. In rodent models, NLRP3-mediated premature immunosenescence drives diabetes-associated vascular ageing through trained-immunity-like mechanisms, linking inflammasome biology not only to acute insulin resistance but to the chronic vascular complications of long-standing T2D [75]. Beyond classical metabolic tissues, NLRP3-driven pyroptotic and cytokine signalling has been documented in diabetic heart failure with preserved ejection fraction, where dysregulated inflammation, oxidative stress, and impaired protein quality control jointly contribute to myocardial dysfunction, illustrating the systemic reach of inflammasome biology beyond glucose homeostasis per se [76].

Metabolite-driven inflammasome activation extends beyond classical lipid and glucose signals. Serum uric acid has been proposed as a mediator of insulin resistance acting in part through inflammasome and oxidative stress pathways, suggesting that purine metabolism represents an additional input into the NLRP3 activation network relevant to T2D [77].

Nutritional modulation of NLRP3-driven metabolic inflammation provides both mechanistic insight and translational opportunity. Omega-3 polyunsaturated fatty acids inhibit NLRP3 inflammasome activation and prevent diet-induced metabolic inflammation in mice, in contrast to the priming effect of saturated fatty acids, indicating that dietary fatty acid composition—not merely caloric load—determines inflammasome activation tone [78,79]. This observation has direct relevance to the broader nutrient-excess framework set out earlier, in which lipid quality rather than quantity alone shapes the downstream innate immune response.

Pharmacological inflammasome inhibition remains at the proof-of-concept stage but is moving toward translational development. The small-molecule NLRP3 inhibitor VTX3232 reduces body weight, hyperglycaemia and associated inflammatory markers in obese mice. This provides causal—though not yet clinical—evidence that NLRP3 activity is a tractable driver of the obese, hyperglycaemic phenotype, and not merely a biomarker of it [17]. The broader clinical pipeline of NLRP3-directed agents, and what it has and has not yet established in humans, is considered in Section 13. Pharmacological NLRP3 blockade in adipose tissue likewise reduces both local inflammation and extracellular matrix remodelling, indicating that inflammasome inhibition addresses both the inflammatory and fibrotic dimensions of adipose tissue dysfunction simultaneously [80]. A broader synthesis of obesity, diabetes, and inflammation has framed NLRP3-IL-1β signalling as one of the most clinically actionable nodes within the immunometabolic network, given the existence of approved IL-1-pathway antagonists in other inflammatory diseases and the mechanistic continuity of their target pathway with T2D pathophysiology [16].

Resolution of inflammation—the active, regulated termination of an inflammatory response—is itself impaired in obesity, and this failure of resolution, and not activation alone, may explain why metaflammation becomes chronic rather than self-limiting; defective resolution pathways leave NLRP3-driven cytokine output unopposed over time [81]. SGLT2 inhibitors, discussed further in Section 13, have also been reported to exert neuroprotective effects plausibly linked to attenuation of inflammasome-driven neuroinflammation, extending the therapeutic relevance of this pathway beyond classical metabolic tissues [82]. Together, the NLRP3–pyroptosis–IL-1β axis represents the most mechanistically direct and pharmacologically tractable link between innate immune activation and the impairment of insulin signalling. It is also where the general mechanism becomes tissue-specific, since the same inflammasome machinery produces markedly different consequences in adipose tissue, islet, and vessel wall.

## 7. Adipose Tissue Macrophages and Tissue-Specific Immune Remodelling

Adipose tissue is the most extensively characterised site of immune-metabolic crosstalk in obesity-associated T2D, and adipose tissue macrophages (ATMs) are its principal cellular effectors. The lean-to-inflammatory transition of the macrophage compartment described in Section 4 acquires, in adipose tissue, a specific spatial organisation whose consequences for tissue remodelling are the subject of this section: with sustained nutrient excess and adipocyte hypertrophy, macrophages accumulate around dying or stressed adipocytes, forming the histological hallmark of obese adipose tissue—the crown-like structure [39,40,41,83]. Two foundational observations remain load-bearing: macrophage content of adipose tissue was shown to scale with adiposity and to account for much of the inflammatory gene expression attributed to fat [84], and diet-induced obesity was subsequently shown to shift the resident population from an alternatively activated state towards a classically activated one, rather than simply to expand a fixed phenotype [85].

This recruitment and phenotypic shift are not a passive bystander process but are actively orchestrated by adipocyte-derived signals. In mice, dysregulation of macrophage prolyl endopeptidase D (PEPD) determines adipose tissue fibro-inflammation and insulin resistance, identifying a specific macrophage-intrinsic enzymatic pathway that links macrophage metabolic state to the fibrotic remodelling that accompanies chronic adipose tissue inflammation [86]. Bioactive lipid mediators generated within stressed adipose tissue similarly recruit and activate macrophages, establishing a direct biochemical link between the lipid excess described earlier and the macrophage-driven inflammation discussed here [87].

ATM heterogeneity extends well beyond the classical M1/M2 polarisation dichotomy. Distinct ATM subpopulations exhibit metabolic reprogramming—including altered glycolytic flux, lipid handling, and ferroptotic susceptibility—that determines their net contribution to local insulin resistance [3]. Macrophage-specific deletion of the ferroptosis regulator Gpx4 alleviates obesity-associated insulin resistance in mice by inducing macrophage ferroptosis, indicating that macrophage cell-death pathways themselves can be therapeutically manipulated to reduce the pro-inflammatory macrophage burden in adipose tissue, on the evidence of a single primary report in mice [88].

Beyond myeloid cells, adipose tissue hosts a complex lymphoid compartment that modulates ATM activation. T-bet-expressing B cells accumulate in obese adipose tissue and exacerbate metabolic dysfunction, in part by skewing the local cytokine milieu toward macrophage activation [89]. Regulatory T cell depletion removes a key restraint on ATM activation and is sufficient to provoke insulin resistance, underscoring that ATM phenotype is determined by the integrated local immune cell network rather than by macrophages in isolation [45].

Macrophage-adipocyte crosstalk operates bidirectionally through both endocrine and paracrine signalling. Leptin, secreted in proportion to adipocyte mass, acts directly on ATMs through JAK2-STAT3 and PI3K-Akt-mTOR signalling to increase their glycolytic flux, phagocytic activity and cytokine secretion, providing a direct mechanistic explanation for how hyperleptinaemia in obesity sustains adipose tissue inflammation [11]. Conversely, ATM-derived cytokines feed back on adipocyte function, suppressing adiponectin secretion and lipid storage capacity, further driving ectopic lipid deposition and reinforcing the nutrient-excess cascade described earlier [26]. The transcription factor NFIA in adipocytes reciprocally regulates mitochondrial and inflammatory gene programmes, providing a single adipocyte-intrinsic node that simultaneously controls the cell’s own bioenergetic capacity and its inflammatory signalling output to surrounding macrophages [25].

Tissue-specific and depot-specific considerations are essential to interpreting ATM biology in T2D. Childhood-onset adipose tissue dysfunction establishes ATM activation patterns that persist into adulthood and may be relatively resistant to later metabolic improvement, implying that the developmental timing of adipose tissue immune remodelling, not only its magnitude, determines long-term metabolic risk [24]. In preclinical models, GLP-1 receptor agonism with tirzepatide acts in part by directly targeting ATMs to reduce obesity-related adipose inflammation, suggesting that part of the metabolic benefit of incretin-based therapies may be mediated through reprogramming of the ATM compartment rather than through weight loss alone [90].

A further dimension of tissue-specific immune remodelling concerns autolysosomal and quality-control pathways within adipocytes themselves. Autolysosomal dysfunction has been implicated in obesity-induced metabolic inflammation, suggesting that failure of adipocyte-intrinsic degradative pathways—distinct from but mechanistically connected to mitophagy failure—contributes to the chronic accumulation of pro-inflammatory signals within expanding adipose tissue [91]. Taken together, the adipose tissue macrophage compartment functions as an integrative sensor and amplifier of local metabolic stress, translating adipocyte-intrinsic dysfunction into a systemic inflammatory signal. What that signal does to mitochondrial function, and through it to the beta cell, determines whether insulin resistance remains compensated.

## 8. Mitochondrial Dysfunction, ROS and Mitophagy

Mitochondrial dysfunction occupies a dual position in the immunometabolic cascade of T2D: it is both a downstream consequence of nutrient excess and inflammatory signalling, and an upstream driver of further innate immune activation, particularly through NLRP3 inflammasome priming (Section 6) [7,92]. Chronic substrate overload—excess glucose and fatty acid flux into the electron transport chain—increases mitochondrial membrane potential and electron leak, generating reactive oxygen species (ROS) in excess of the cell’s antioxidant buffering capacity [93,94]. This oxidative stress is not confined to a single tissue: it has been documented across adipose tissue, skeletal muscle, liver, pancreatic islets, and circulating immune cells in T2D, and its severity correlates with both the degree of insulin resistance and markers of systemic inflammation [95,96].

In skeletal muscle, mitochondrial dysfunction directly impairs insulin-stimulated glucose disposal through ROS-dependent activation of stress kinases (notably JNK) that interfere with insulin receptor substrate signalling, in addition to non-mitochondrial ROS sources that contribute independently to this impairment [97]. This mechanism provides a direct molecular continuity between the skeletal muscle inflammation described earlier and the insulin-signalling defects elaborated below, with mitochondrial ROS acting as a shared upstream input to both.

Autophagy and its specialised mitochondrial form, mitophagy, constitute the principal quality-control mechanism opposing the accumulation of damaged, ROS-generating mitochondria. In obesity and T2D, autophagic and mitophagic flux are impaired, permitting the accumulation of dysfunctional mitochondria that serve as a persistent source of ROS and, importantly, of mitochondrial DNA and other damage-associated molecular patterns capable of directly priming the NLRP3 inflammasome [98,99]. Mice deficient in the mitophagy receptor FUNDC1 show impaired mitochondrial quality and aggravated diet-induced metabolic dysfunction, providing direct genetic evidence that intact mitophagy is necessary to limit diet-induced inflammatory and metabolic injury [99]. Mitophagy-mediated adipose inflammation has been linked specifically to impaired hepatic insulin signalling, establishing mitophagy failure in adipose tissue as a node with inter-organ, not merely local, metabolic consequences [32].

Mitochondrial DNA escaping into the cytosol is sensed by a second pathway that operates in parallel to the inflammasome and has been comparatively neglected in the metabolic literature. Cyclic GMP-AMP synthase (cGAS) binds cytosolic double-stranded DNA irrespective of its origin and synthesises 2′3′-cyclic GMP-AMP, which activates the endoplasmic reticulum adaptor STING and, through TBK1 and IRF3, induces type I interferon and NF-κB-dependent transcription [100]. Because impaired mitophagy is precisely the condition under which mitochondrial DNA reaches the cytosol, the same quality-control failure described above feeds two distinct danger-sensing systems rather than one. Genetic evidence supports a causal role in metabolic disease: loss of the adipocyte protein DsbA-L increases mitochondrial DNA release and activates cGAS-STING, producing obesity-associated inflammation and insulin resistance in mice, whereas STING deletion protects against them [101]. In the liver, STING signalling in Kupffer cells promotes progression of non-alcoholic fatty liver disease, extending the pathway to the hepatic arm of the network [102]. Whether cGAS-STING contributes comparably in human T2D is untested, and the pathway’s physiological role in antiviral defence complicates its therapeutic suppression; it is included here because it identifies a second, mechanistically distinct consequence of the mitophagy defect that any account restricted to NLRP3 would miss.

Advanced glycation end-products (AGEs), generated under conditions of chronic hyperglycaemia, provide an additional route by which sustained metabolic stress damages mitochondrial–insulin signalling crosstalk. AGEs impair the PI3K/Akt/mTOR insulin signalling pathway and contribute to insulin resistance, in part through AGE receptor-mediated oxidative stress that further burdens already-compromised mitochondrial function [103]. This mechanism is particularly relevant to tissues exposed to high local glucose flux and helps explain why glycaemic variability itself, independent of mean glucose, can aggravate the immunometabolic phenotype.

Inter-organ miscommunication mediated by mitochondrial dysfunction has been proposed as a unifying mechanism in T2D progression, in which mitochondrial dysfunction in one tissue generates circulating signals (including mitochondria-derived peptides and metabolites) that propagate metabolic stress to other tissues, including the endocrine pancreas [92]. This concept is consistent with the broader inter-organ crosstalk framework discussed below and reinforces that beta-cell mitochondrial dysfunction in T2D should not be considered solely a beta-cell-intrinsic phenomenon.

Sarcopenic obesity provides a clinically important illustration of the tissue-specific consequences of combined mitochondrial dysfunction and chronic inflammation: the co-occurrence of reduced muscle mass and quality with excess adiposity is associated with disproportionately severe cardiovascular and metabolic risk. This plausibly reflects a compounded mitochondrial and inflammatory burden in skeletal muscle that exceeds the sum of either process alone [104]. Lipotoxicity itself exerts much of its damaging effect through mitochondrial overload and dysfunction rather than through lipid accumulation per se, reinforcing mitochondrial health as the proximate determinant of whether ectopic lipid is metabolically inert or actively pathogenic [19].

Cardiovascular tissue is a particularly sensitive target of this mitochondrial–inflammatory axis: molecular mechanisms underlying T2D-related heart disease converge substantially on cardiomyocyte mitochondrial dysfunction and oxidative stress, continuous with the inflammasome-driven cardiac pathology described above [105]. Diabetic cardiomyopathy specifically has been framed around oxidative stress as a central molecular mechanism with identifiable emerging therapeutic targets, reinforcing that the mitochondrial–ROS axis described here is not confined to systemic insulin sensitivity but extends directly into a major, clinically distinct diabetic complication [106]. More broadly, oxidative stress has been positioned as a mechanistic bridge between T2D pathophysiology and its downstream cardiovascular complications, with lifestyle modification proposed as a route to favourably modulate this oxidative burden alongside pharmacological approaches [107]. Gestational diabetes similarly exhibits a cellular and molecular pathophysiology substantially overlapping with that of T2D, including prominent mitochondrial dysfunction and oxidative stress components, suggesting that pregnancy represents a physiological stress test that can unmask an underlying immunometabolic susceptibility shared with T2D [108].

In sum, mitochondrial dysfunction, ROS generation and impaired mitophagy together constitute a self-sustaining node within the immunometabolic network that is at once a consequence of upstream metaflammation and nutrient excess (Section 3 and Section 4) and a direct trigger of NLRP3 inflammasome activation (Section 6). Nowhere is that node more consequential than in the pancreatic beta cell, whose oxidative defences are among the weakest of any endocrine tissue.

## 9. Beta-Cell Immunometabolism

Pancreatic beta-cell dysfunction is the event that converts compensated insulin resistance into overt T2D along the route traced here, and its immunometabolic basis in that setting is now well established. In the insulin-deficient and lean phenotypes considered in Section 11, this node is entered earlier and with substantially less upstream adipose input. Islets are not immunologically privileged: under metabolic stress, resident and infiltrating macrophages, alongside other immune cells, actively shape islet function and survival, such that beta-cell failure should be understood as an immunometabolic endpoint and not as an isolated secretory defect [109,110].

Chronic exposure to elevated glucose and free fatty acids—glucolipotoxicity—directly impairs beta-cell function through several converging mechanisms: induction of endoplasmic reticulum stress, generation of reactive lipid species, and activation of pro-apoptotic signalling cascades [111,112]. Lipotoxicity-induced beta-cell dysfunction specifically involves disruption of lipid handling pathways within the beta cell, generating toxic lipid intermediates that impair insulin secretory capacity and ultimately trigger beta-cell apoptosis [111]. The 12-lipoxygenase pathway has been identified as a specific route by which lipid metabolites contribute to immunoinflammatory beta-cell destruction, linking islet lipid metabolism directly to local inflammatory signalling [112].

Islet inflammation in T2D is characterised by increased local cytokine production (notably IL-1β, consistent with the NLRP3 mechanisms discussed in Section 6), macrophage infiltration, and chemokine-mediated recruitment of additional immune cells [113,114]. Chemokines have been specifically identified as an emerging mechanistic node in pancreatic islet inflammation, providing a molecular explanation for the progressive, self-amplifying recruitment of immune cells into stressed islets over the course of T2D progression [114].

Pancreatic fatty infiltration—ectopic lipid deposition within and around islets, directly analogous to ectopic lipid deposition in liver and skeletal muscle—has been specifically associated with islet inflammation and hyperglycaemia in T2D. The islet microenvironment is therefore subject to the same glucolipotoxic and inflammatory mechanisms operating systemically [115]. Intrapancreatic adipocytes have furthermore been linked to beta-cell dedifferentiation in human T2D, suggesting that loss of mature beta-cell identity, and not beta-cell death alone, is an important and potentially reversible contributor to the secretory failure observed clinically. That association has so far been documented in a single primary study of human tissue [116].

Macrophages occupy an ambivalent position in islet immunometabolism, capable of either supporting islet homeostasis or driving its failure depending on activation state and disease stage—a duality captured in the framing of macrophages as “friend or foe” in diabetes pathogenesis and therapy [117]. A specific protective mechanism illustrating this duality involves the islet regeneration protein Reg3g, which promotes macrophage-mediated clearance of dysfunctional, damaged beta-cell mitochondria, linking the mitochondrial quality-control mechanisms described above directly to a beneficial, pro-regenerative islet macrophage function. This mechanism has been reported in a single recent study and awaits independent confirmation [118]. This finding indicates that therapeutic strategies might usefully aim not to eliminate islet macrophages broadly but to shift their functional programme toward this regenerative, clearance-promoting phenotype.

Beta-cell failure in T2D should also be situated within the broader framework of inter-organ crosstalk: islet dysfunction develops in the context of, and is substantially driven by, signals arriving from insulin-resistant peripheral tissues. These include circulating free fatty acids, inflammatory cytokines and altered incretin signalling. Islet failure is therefore not an independent, beta-cell-autonomous process [119,120]. This framing is consistent with the comprehensive classification of T2D pathogenesis, mediators, and complications, which positions islet inflammation as one node within a multi-organ inflammatory network rather than a terminal, isolated event [121,122].

Clinically, the trajectory from islet inflammation to overt T2D unfolds over years, with islet inflammatory markers detectable before the onset of frank hyperglycaemia, paralleling the early inflammatory trajectory of systemic insulin resistance described earlier [110]. The broader gut-related axes affecting T2D, including incretin signalling and microbial metabolite exposure, also converge on beta-cell function—the last major input into the network still to be accounted for [123].

## 10. Gut-Derived Signals, Metabolic Endotoxaemia and Microbial Metabolites

The gastrointestinal tract and its resident microbiota constitute a major, systemically influential input into the immunometabolic network underlying obesity-associated T2D. Under conditions of nutrient excess and dysbiosis, intestinal barrier integrity is compromised, permitting translocation of microbial components—most notably lipopolysaccharide (LPS)—into the systemic circulation, a state termed metabolic endotoxaemia [34,124]. The term originates from two observations in mice: high-fat feeding raises plasma LPS two- to threefold, and continuous subcutaneous LPS infusion at a comparable concentration is by itself sufficient to reproduce weight gain, hepatic steatosis and insulin resistance. Gut-derived endotoxin is therefore a cause of the phenotype and not only a marker of it [125]. Circulating LPS engages TLR4 on macrophages and other innate immune cells in adipose tissue and liver, directly activating the same NF-κB-dependent inflammatory signalling cascades primed by nutrient excess (Section 3, Section 4, Section 5 and Section 6), thereby providing a parallel, microbiota-derived route into the core inflammatory pathway of T2D [126].

Gut dysbiosis in T2D is characterised by reproducible shifts in microbial community composition, generally featuring reduced microbial diversity and an altered ratio of major bacterial phyla, alongside reduced production of beneficial microbial metabolites [127]. Short-chain fatty acids (SCFAs), generated by microbial fermentation of dietary fibre, exemplify the protective arm of this axis: dietary fibre intake increases SCFA production, which in turn favourably modulates lipid profiles and reduces inflammatory markers in patients with metabolic disease. This is a direct, diet-modifiable route by which the gut microbiota can dampen the inflammatory cascade instead of amplifying it [128].

Bile acids constitute a second major class of microbiota-modulated signalling molecules relevant to T2D immunometabolism. Acting through the nuclear farnesoid X receptor and the membrane receptor TGR5, bile acids coordinately regulate both metabolic and inflammatory signalling, and their composition is substantially shaped by microbial metabolism. This provides a mechanistic link between microbiota composition and systemic inflammatory and metabolic tone, distinct from the LPS-TLR4 axis but complementary to it [129].

Specific microbial taxa have been identified with direct, mechanistically characterised effects on host metabolic inflammation. A purified membrane protein from *Akkermansia muciniphila*, and the pasteurised bacterium itself, improve metabolic parameters in obese and diabetic mice, providing one of the most direct examples of a single, well-characterised microbial component capable of favourably modulating the gut–immune–metabolic axis [130]. This finding has since been carried into humans: in a proof-of-concept randomised study in overweight and insulin-resistant volunteers, pasteurised *A. muciniphila* was safe and tolerable and improved insulin sensitivity and plasma markers of hepatic and metabolic health, though the trial was small and not powered for glycaemic endpoints [131]. Synbiotic interventions, combining probiotics and prebiotics, have shown superior efficacy compared with probiotics alone in improving glycaemic parameters in randomised controlled trials in T2D, providing translational, human-level evidence for the causal relevance of microbiota composition to glycaemic control, beyond purely mechanistic or animal-model evidence [132].

Bacteraemia risk is itself measurably elevated in T2D, a clinically important corollary of gut barrier dysfunction and metabolic endotoxaemia that links the mechanistic gut–immune axis directly to infectious morbidity in this population [133].

Diet–gut microbiota–epigenetic interactions provide an additional layer of mechanisms, linking dietary exposure through microbiota remodelling to durable epigenetic changes in host metabolic and immune gene expression, suggesting that microbiota-mediated signals may contribute to the kind of persistent, trained-immunity-like inflammatory states described earlier [134]. Traditional herbal formulations have been reported to improve metabolic and vascular parameters through gut microbiota-dependent and macrophage-polarisation-dependent mechanisms; this evidence is preclinical and is noted here only as an indication that the axis is pharmacologically tractable [135,136].

Comprehensive gut microbiome modulation strategies—combining nutritional and pharmacological approaches—are increasingly framed as adjunctive T2D therapeutics in their own right and not merely as incidental modifiers of metabolic disease, reflecting the maturation of this mechanistic axis into a distinct therapeutic target, discussed further in Section 13 [137,138]. The rationale for this positioning is that the microbiome acts on systemic innate immune tone rather than on a single metabolic pathway, which is what makes it a pharmacological target in its own right [139]. This strategy has been elaborated in detail for the closely related setting of metabolic dysfunction-associated steatotic liver disease (MASLD), where prebiotics, probiotics, postbiotics and faecal microbiota transplantation have each been proposed as graduated nutritional interventions targeting the gut microbiome. The resulting tiered framework offers an analogy for the gut–liver–pancreas axis in T2D, given the substantial mechanistic overlap between the two conditions already discussed. It would nonetheless require validation in T2D before being transferred [140]. Overall, the gut microbiota–immune axis functions as a systemic amplifier or attenuator of the metaflammatory state established in adipose tissue, interacting with—rather than operating independently of—the core innate immune and inflammasome pathways already described.

The chain has now been traced across nutrient excess and metaflammation, innate immune activation, NLRP3-driven cytokine signalling, adipose tissue macrophage remodelling, mitochondrial dysfunction, beta-cell immunometabolism and gut-derived systemic input. Table 2 summarises the principal mechanisms, cell and tissue substrates, and molecular mediators discussed across these sections. Figure 1 illustrates their integration into a single immunometabolic network linking nutrient excess to insulin resistance, beta-cell dysfunction and chronic low-grade inflammation.

## 11. Heterogeneity of Type 2 Diabetes and the Limits of a Linear Model

The chain traced in Section 3, Section 4, Section 5, Section 6, Section 7, Section 8, Section 9 and Section 10 is presented as a sequence—nutrient excess, innate immune activation, inflammasome-driven cytokine signalling, tissue-specific immune remodelling, mitochondrial failure and beta-cell decompensation—because that is the order in which its individual links have been experimentally established. The sequence should not, however, be mistaken for the disease. It describes with reasonable fidelity the obesity-associated route into T2D, which accounts for the majority of cases in high-income populations and for very nearly all of the mechanistic literature synthesised here [5], but T2D is not a single pathophysiological entity, and several of its recognised forms enter this network at a different point, or bypass parts of it altogether.

Data-driven subgrouping has made that heterogeneity concrete. Cluster analysis of six routinely measured variables in 8980 patients with newly diagnosed adult-onset diabetes resolved five reproducible subgroups with distinct trajectories and complication profiles: severe autoimmune diabetes, severe insulin-deficient diabetes, severe insulin-resistant diabetes, mild obesity-related diabetes, and mild age-related diabetes [141]. Only two of these—severe insulin-resistant and mild obesity-related diabetes—correspond closely to the adipose-centred mechanism developed above. Severe insulin-deficient diabetes is characterised by early beta-cell failure in the relative absence of obesity and of marked insulin resistance, while mild age-related diabetes, the largest of the clusters, follows a comparatively indolent course in which neither adipose inflammation nor beta-cell destruction is prominent. A reanalysis of 2316 newly diagnosed Chinese and 685 United States participants, reported as a correspondence rather than as a full report, recovered four of these subgroups—the severe autoimmune cluster was not resolvable in those cohorts—in closely similar proportions in the two populations, severe insulin-deficient diabetes accounting for 13.5% and 14.3% and severe insulin-resistant diabetes for 8.6% and 7.9%, respectively [142]. What that reanalysis establishes is the reproducibility of the partition beyond its European derivation cohort, rather than a systematic shift in subgroup boundaries by ancestry; whether the relative weight of the mechanisms described in this review differs between populations is a question these data leave open.

The lean and normal-weight phenotype is the clearest case in which the account given here does not apply in its usual order. Between 3% and 8% of T2D worldwide occurs in individuals whose body mass index falls within the normal range, with marked clustering in South Asian, sub-Saharan African and Caribbean populations. It has been characterised as driven by an early and severe insulin secretory deficit, not by the adipose expansion, macrophage infiltration and systemic metaflammation with which the present account opens [143]. That contrast is drawn, however, in a narrative synthesis with two limitations. The phenotype is defined largely by body mass index and by outcome, not by standardised measurement of insulin secretion and sensitivity, and the evidence for predominant beta-cell failure is itself described as limited. The mechanistic distinction should therefore be read as a hypothesis, not as an established property of the phenotype. The body mass index thresholds that define it also require ancestry-specific cut-offs, which the available studies do not apply uniformly. For these patients the metaflammatory sequence is not the initiating event, and the beta-cell immunometabolism described in Section 9 may operate with comparatively little of the upstream adipose input described in Section 3, Section 4 and Section 7. Whether the intra-islet inflammatory mechanisms are the same in the absence of systemic metaflammation, or whether they are engaged by a different set of upstream signals, has not been established, and it is a question on which the therapeutic inferences of this review directly depend.

Genetic evidence supports this partition directly, not by inference. Multi-ancestry meta-analysis has resolved the polygenic architecture of T2D into mechanistic clusters that map onto separable pathophysiological processes—beta-cell dysfunction with and without a proinsulin signature, obesity, lipodystrophy, and liver lipid metabolism among them—and the cluster-specific genetic burden carried by an individual has been associated with a different pattern of vascular outcome [144,145]. These are associations established at the level of large populations; their capacity to allocate treatment in an individual patient has not been demonstrated. The implication for the framework advanced here is specific rather than general: the mechanisms synthesised in Section 3, Section 4, Section 5, Section 6, Section 7, Section 8, Section 9 and Section 10 are unlikely to contribute equally in every patient, and the node that predominates plausibly differs between genetically defined subgroups in a manner that is in principle measurable rather than merely postulated, though not yet validated as a basis for treatment allocation.

Three qualifications follow for the reading of this review. First, the arrows in Figure 1 represent the dominant flow in obesity-associated disease and not an obligatory sequence: in the insulin-deficient and lean phenotypes the beta-cell node is entered early and comparatively directly. Second, the network is in any case not linear at the level of the individual mechanism—mitochondrial stress both follows and precedes inflammasome activation, trained immunity outlasts the stimulus that induced it, and gut-derived signals operate in parallel rather than in series—so that what is contingent is the tissue-to-tissue ordering rather than the mechanisms themselves. Third, the therapeutic inferences of Section 13 are correspondingly conditional: an inflammasome-directed agent would be expected to act, if at all, in patients whose disease is driven by the adipose-inflammatory node, and that is precisely the stratification which the endotyping efforts discussed in Section 14 have not yet delivered [146]. This supplies a further reading of the CANTOS result alongside those set out in Section 6: a trial recruiting on an inflammatory rather than an immunometabolic criterion will enrol a mechanistically heterogeneous population, within which a subgroup-restricted benefit would be diluted towards the null.

The framework advanced in this review should therefore be understood not as a general model of type 2 diabetes, but as a mechanistic account of its obesity-associated, inflammatory form, whose extension to the other recognised subgroups is a question to be tested rather than a step to be assumed. Table 3 summarises the principal recognised sources of heterogeneity, their relation to the mechanistic chain developed in Section 3, Section 4, Section 5, Section 6, Section 7, Section 8, Section 9 and Section 10, and the hypotheses that each generates for immunometabolic targeting.

## 12. Molecular Pathways: AMPK, mTOR, PI3K/AKT, NF-κB, JNK and Sirtuins

The tissue- and cell-type-specific mechanisms described in the preceding sections converge on a relatively small set of intracellular signalling nodes that function as integrators of nutrient, energy, and inflammatory status. These pathways provide the molecular grammar that explains why metabolic and inflammatory signals are so tightly coupled across virtually every cell type discussed in this review.

AMP-activated protein kinase (AMPK) functions as the cell’s principal energy sensor, activated by a rising AMP:ATP ratio under conditions of energy deficit, and exerts broadly anti-inflammatory, insulin-sensitising effects through phosphorylation of targets that inhibit NF-κB signalling and promote fatty acid oxidation over lipogenesis [147]. Pharmacological AMPK activation—most notably by metformin, discussed further in Section 13—recapitulates many of these beneficial effects, providing a direct mechanistic bridge between energy-sensing biology and clinical glucose-lowering therapy [148].

The mechanistic target of rapamycin (mTOR), in contrast, is activated by nutrient sufficiency (amino acids, growth factors) and functions largely in opposition to AMPK, promoting anabolic growth programmes but also, under chronic nutrient excess, contributing to inflammatory signalling and insulin resistance through feedback inhibition of insulin receptor substrate proteins downstream of chronic mTORC1 activation [149]. Natural mTOR inhibitors have accordingly been explored as a therapeutic strategy in T2D, explicitly framed as a means of correcting the chronic nutrient-sensing imbalance that mTOR hyperactivation represents in this disease [149].

PI3K/Akt signalling is the principal proximal effector of canonical insulin receptor signalling, transducing receptor activation into GLUT4 translocation, glycogen synthesis, and broader anabolic and survival signalling, and its impairment by inflammatory serine kinases (JNK, IKKβ) is the most direct molecular lesion explaining cytokine-induced insulin resistance described throughout Section 4, Section 5, Section 6, Section 7 and Section 8 [20]. PI3K/Akt signalling is not confined to canonical insulin receptor engagement. The same pathway operates downstream of leptin receptor activation in macrophages and of multiple cytokine receptors, so that PI3K/Akt serves as a hub shared by metabolic and immune receptor systems. That shared architecture helps explain why metabolic and immune signals are so readily cross-wired in chronically stressed tissues [11].

NF-κB signalling is the master transcriptional regulator of the inflammatory gene programme. Virtually every upstream signal discussed in this review converges on it: TLR ligands, cytokine receptors, ROS and ER stress alike. NF-κB activity is in turn regulated by core metabolic processes and regulates them in return, so the relationship between inflammatory transcription and metabolic state is bidirectional, not one-way [150]. This mutual regulation explains why interventions that reduce metabolic substrate flux (caloric restriction, exercise, AMPK activators) reliably reduce NF-κB-dependent inflammatory tone even in the absence of a directly anti-inflammatory mechanism of action.

JNK signalling functions as a key stress-activated kinase node downstream of ROS, ER stress, and inflammatory cytokine receptors, and is mechanistically central to insulin receptor substrate serine phosphorylation across adipose tissue, liver, and skeletal muscle [42]. The genetic evidence is unusually clean for this pathway: Jnk1-deficient mice are protected against diet-induced obesity and insulin resistance, with reduced inhibitory IRS-1 serine phosphorylation, establishing JNK as necessary for the phenotype rather than merely associated with it [151]. At the molecular level, JNK phosphorylates IRS-1 at Ser307, a residue adjacent to the phosphotyrosine-binding domain, and this modification blocks the interaction between IRS-1 and the juxtamembrane region of the insulin receptor—the most precisely defined lesion linking an inflammatory kinase to interrupted insulin signalling [152].

Sirtuins, and sirtuin 1 (SIRT1) in particular, form the principal counter-regulatory arm to these inflammatory pathways. As NAD+-dependent deacetylases they promote mitochondrial biogenesis, suppress NF-κB-dependent transcription and improve insulin sensitivity. SIRT1 activity is characteristically reduced in T2D, and its restoration has been proposed as a unifying therapeutic strategy, since it addresses the mitochondrial and inflammatory arms of the disease at once [153]. Exercise-intervention mechanistic syntheses in T2D also place the AMPK/SIRT1/PGC-1α axis within a modifiable programme of mitochondrial biogenesis, lipid oxidation, and anti-inflammatory myokine signalling [154]. The upstream triggers, downstream effects, and disease relevance of these core signalling nodes—AMPK, mTOR, PI3K/AKT, NF-κB, JNK, sirtuins, and NLRP3-caspase-1—are summarised in Table 4.

These pathways do not operate as isolated linear cascades but as an interconnected signalling network whose net output—pro-inflammatory and catabolic, or anti-inflammatory and insulin-sensitising—is determined by the integrated balance of AMPK/SIRT1 activity against mTOR/NF-κB/JNK activity at a given moment in a given tissue. This integration explains the pleiotropic, multi-tissue effects of several therapeutic agents discussed in the next section, whose mechanisms of action engage multiple nodes of this network simultaneously rather than a single isolated target [155,156]. Hypothalamic microglial activation shows the same convergence operating outside classical peripheral metabolic tissue. Microglial NF-κB and inflammatory signalling in the hypothalamus contribute to central insulin resistance and to disordered regulation of energy balance. The network therefore extends across the full range of tissues relevant to T2D pathophysiology, the central nervous system included [156].

## 13. Therapeutic Targets and Pharmacological Implications

**Figure 2:**
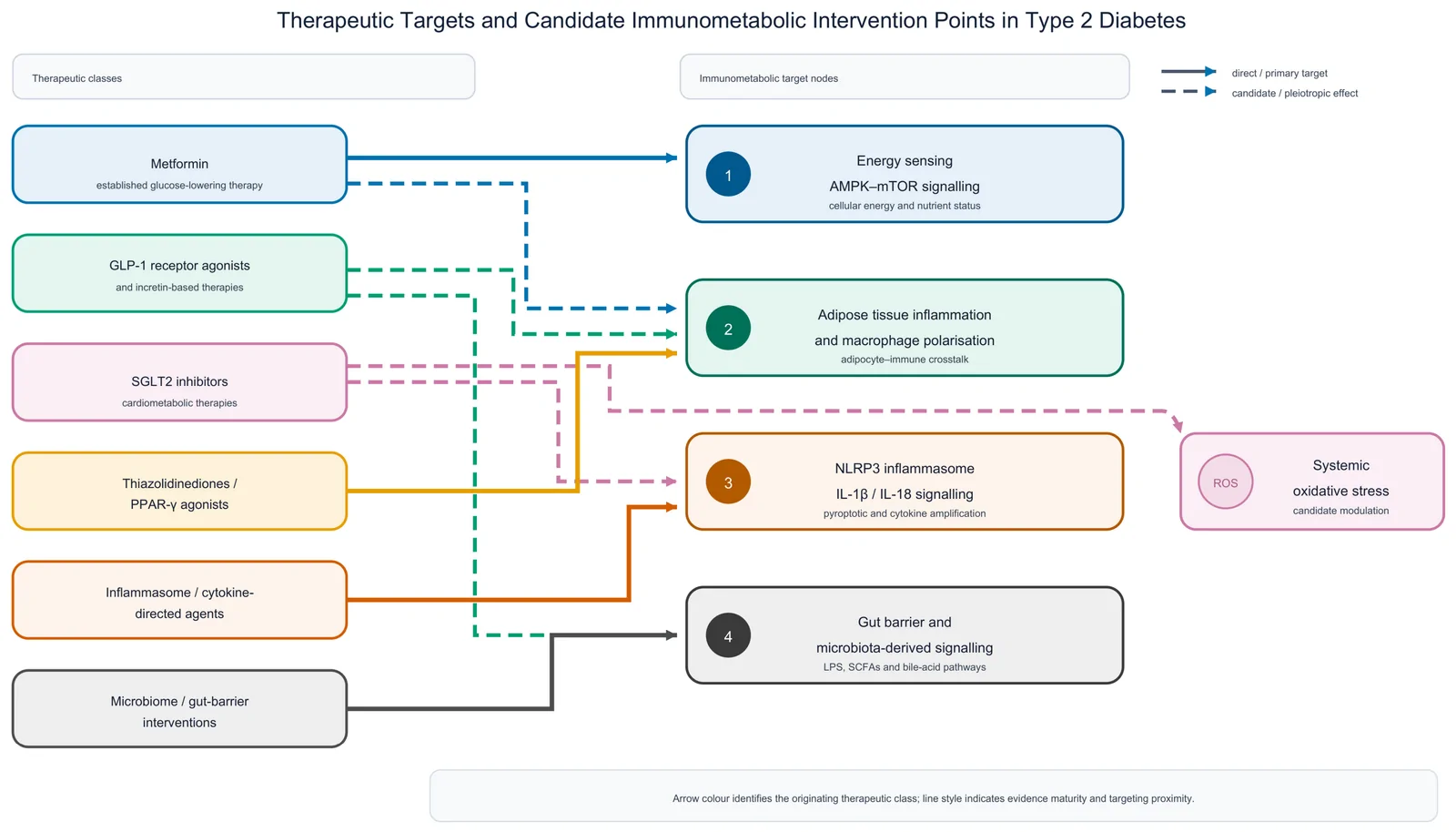
Therapeutic targets and candidate immunometabolic intervention points in type 2 diabetes (T2D). Established and emerging therapeutic classes—metformin, glucagon-like peptide-1 (GLP-1) receptor agonists, sodium–glucose cotransporter 2 (SGLT2) inhibitors, thiazolidinediones/peroxisome proliferator-activated receptor-γ (PPAR-γ) agonists, inflammasome/cytokine-directed agents, and microbiome/gut-barrier interventions—are mapped onto four core immunometabolic nodes: energy sensing/AMP-activated protein kinase (AMPK)–mechanistic target of rapamycin (mTOR) signalling; adipose-tissue inflammation/macrophage polarisation; NLRP3 inflammasome/IL-1β/IL-18 signalling; and gut-barrier/microbiota-derived signalling. Solid arrows denote direct or primary targeting and dashed arrows candidate or pleiotropic immunometabolic modulation. Arrow colour identifies the originating therapeutic class, but is redundant with the geometry of the path: every arrow originates at the labelled box of its class and can be followed without reference to colour. The palette follows the Okabe–Ito colour-vision-deficiency-safe scheme with six classes; however, complete separation under simulated deuteranopia is not achievable by colour alone. Abbreviations: T2D, type 2 diabetes; GLP-1, glucagon-like peptide-1; SGLT2, sodium–glucose cotransporter 2; PPAR-γ, peroxisome proliferator-activated receptor-γ; AMPK, AMP-activated protein kinase; mTOR, mechanistic target of rapamycin; NLRP3, NOD-, LRR- and pyrin domain-containing protein 3; IL-1β, interleukin-1β; IL-18, interleukin-18; ROS, reactive oxygen species; LPS, lipopolysaccharide; SCFAs, short-chain fatty acids. The map summarises mechanistic targeting and not demonstrated glycaemic efficacy: as discussed in Section 13, the clinical evidence for inflammasome- and cytokine-directed agents currently supports cardiovascular rather than glycaemic benefit. The figure was prepared by the authors using Microsoft PowerPoint (Microsoft Corporation, Redmond, WA, USA) and Inkscape v1.3 (Inkscape Project, free and open-source vector graphics software; https://inkscape.org, accessed on 20 July 2026).

The mechanistic chain described in Section 3, Section 4, Section 5, Section 6, Section 7, Section 8, Section 9, Section 10, Section 11 and Section 12—nutrient excess, innate immune activation, NLRP3-driven cytokine signalling, tissue-specific immune remodelling, mitochondrial dysfunction, and beta-cell failure—provides a rational framework for understanding both the established and emerging pharmacology of T2D. At the end of this section, Table 5 summarises the primary metabolic actions, candidate immunometabolic effects, molecular targets, and evidence strength of the main therapeutic classes discussed below, and Figure 2 maps these classes onto the specific immunometabolic intervention points they engage.

Metformin remains the most extensively studied agent at the intersection of glucoregulatory and immunomodulatory mechanism. Its principal glucoregulatory action involves AMPK-dependent suppression of hepatic gluconeogenesis, but a substantial body of evidence indicates that metformin additionally exerts direct anti-inflammatory effects, including modulation of macrophage polarisation in adipose tissue, independent of its glucose-lowering action [148,157]. Metformin has been specifically characterised as a multifaceted regulator of adipose tissue metabolism, inflammation, and regenerative capacity, directly engaging the ATM biology already described rather than acting solely on hepatocytes [158]. This local immunomodulatory action is consistent with reports that metformin and related glucose-lowering agents influence wound healing through modulation of inflammatory and immune cell activity at the tissue level, a peripheral but mechanistically congruent corollary of its adipose- and macrophage-directed effects [159].

GLP-1 receptor agonists exemplify a drug class whose anti-inflammatory and immunomodulatory actions are increasingly understood as integral, rather than incidental, to their metabolic benefit. The reprogramming of adipose tissue macrophages by tirzepatide described in Section 7 is mechanistically distinct from, but likely additive with, exercise-induced myokine and AMPK signalling: combining exercise with GLP-1 receptor agonist treatment produces a greater reduction in metabolic syndrome severity than either intervention alone, consistent with two independent anti-inflammatory mechanisms converging on the same underlying immunometabolic network [160]. Tissue-level clinical observations in patients with T2D offer indirect corroboration that this anti-inflammatory action extends beyond glycaemic control per se, although the causal mechanism at each tissue remains to be resolved [161,162]. Neuroprotective effects of GLP-1 receptor agonists have also been documented in animal models of diabetes, plausibly reflecting attenuation of the central inflammatory mechanisms described earlier, though direct evidence in human brain tissue is lacking [163]. Newer oral, non-peptide agonists such as orforglipron extend this mechanistic class to an oral formulation with clinically meaningful metabolic effects in early trials, though immunometabolic data for this specific agent are not yet available [164]. One safety consideration warrants mention without overstating its mechanistic basis: the potential sarcopenic risk associated with GLP-1 receptor agonist-induced weight loss, which bears directly on the sarcopenic obesity phenotype discussed in Section 8 [155,165].

SGLT2 inhibitors constitute a third major drug class with direct, mechanistically grounded anti-inflammatory action, distinct from their primary glucosuric mode of action. SGLT2 inhibitors have been reported to exert anti-inflammatory effects on macrophages, attenuating the same pro-inflammatory macrophage activation programmes already described [166]. Neuroprotective effects of SGLT2 inhibitors have also been reported, plausibly reflecting reduction in systemic and possibly central inflammatory tone [82].

Direct inflammasome and cytokine-pathway targeting represents the most mechanistically proximate translational strategy emerging from the NLRP3 biology discussed in Section 6. The NLRP3 inhibitor VTX3232 reduces body weight, hyperglycaemia, and inflammatory markers in preclinical models, representing one of the clearest examples of a therapeutic agent designed explicitly around the mechanistic pathway elaborated in this review, with glycaemic control as a secondary consideration [17]. The clinical pipeline is nonetheless broader than a single preclinical compound. The orally available NLRP3 inhibitor dapansutrile (OLT1177) has completed early human studies in two inflammatory indications. The first was an open-label, dose-adaptive, proof-of-concept phase 2a trial in acute gout flares: target-joint pain decreased from baseline in all four dose groups, from 100 mg to 2000 mg daily, in an uncontrolled design that does not support a causal inference [167]. The second was a phase 1B dose-escalation study in 30 patients with New York Heart Association class II–III systolic heart failure. Up to 14 days of treatment was safe and well tolerated, and the 2000 mg cohort showed improvements in left ventricular ejection fraction and in exercise time, on a design that included only two placebo cases per cohort and was not powered for efficacy [168]. That second study also reported the only human metabolic signal so far available for this agent: median fasting plasma glucose fell by 10.5 mg/dL from baseline across the pooled dapansutrile-treated patients (*p* = 0.021) and by 32.5 mg/dL in the 13 treated patients who had T2D (*p* = 0.029). These were exploratory within-group comparisons rather than contrasts against placebo, were unadjusted for multiplicity, and reached significance in no individual dose cohort; they generate a hypothesis rather than establish an effect. These studies establish that systemic NLRP3 inhibition is feasible and tolerable in humans with cardiometabolic disease, but neither was designed to assess glycaemia, and no NLRP3-directed agent has to date demonstrated a glucose-lowering effect against a control arm in a human trial. More broadly, the pathophysiological and clinical case for targeting obesity-associated inflammation directly—rather than treating it as a secondary consequence of hyperglycaemia—has been articulated as a coherent therapeutic strategy distinct from, and complementary to, conventional glucose-lowering approaches [16]. That case, however, must be read against the trial evidence set out in Section 6: IL-1β neutralisation reduced cardiovascular events without preventing incident diabetes [71,72], so an agent’s capacity to lower inflammatory markers cannot be assumed to translate into glycaemic benefit. Colchicine illustrates the same asymmetry from a different direction. Low-dose colchicine reduces recurrent cardiovascular events both after myocardial infarction and in chronic coronary disease [169,170], confirming that broad anti-inflammatory therapy modifies outcomes across the cardiovascular–kidney–metabolic continuum, yet it has not demonstrated meaningful glucose-lowering activity. The consistent pattern across these trials is that anti-inflammatory intervention in this disease space has so far proved more effective against vascular endpoints than against glycaemia—a constraint that any inflammasome-directed development programme in T2D will need to address explicitly, most plausibly through earlier intervention or tissue-selective delivery.

Conversely, the two agent classes with the strongest cardiometabolic outcome data do lower inflammatory tone measurably in humans, which strengthens the mechanistic argument advanced throughout this review. In the SELECT trial, semaglutide reduced cardiovascular events in overweight and obese patients without diabetes, and was accompanied by a 37.8% reduction in high-sensitivity C-reactive protein at 104 weeks. Only part of that fall is attributable to weight loss: it was already evident at 4 to 8 weeks, before major weight loss had occurred, was present also among participants who did not lose weight, and was independent of low-density lipoprotein cholesterol and statin use [171,172]. Whether this reflects direct engagement of the adipose tissue macrophage compartment, reduced substrate flux, or both cannot be resolved from outcome trials alone, and dedicated immunometabolic endpoints in human tissue remain the principal missing evidence.

Gut microbiome-directed therapeutic strategies, introduced mechanistically in Section 10, are increasingly positioned as adjunctive T2D treatments, combining nutritional (prebiotic, dietary fibre, synbiotic) and microbiota-targeted pharmacological approaches [132,137]. Natural compounds with pleiotropic anti-inflammatory and insulin-sensitising actions, such as quercetin, have also been investigated as adjunctive agents engaging multiple nodes of the signalling network described above, including AMPK and NF-κB pathways, although their translational maturity remains considerably behind that of metformin, GLP-1 receptor agonists, and SGLT2 inhibitors [173].

Thiazolidinediones warrant more than passing mention here, because they appear at first to contradict the argument of this review: they are, mechanistically, anti-inflammatory insulin sensitisers, and unlike IL-1β neutralisation they lower glucose. In the ACT NOW trial, pioglitazone reduced conversion from impaired glucose tolerance to diabetes from an annual incidence of 7.6% to 2.1%, a hazard ratio of 0.28 (95% CI 0.16–0.49; *p* < 0.001) [174]. IRIS enrolled 3876 patients without diabetes who had insulin resistance and a recent ischaemic stroke or transient ischaemic attack; there pioglitazone reduced both the primary vascular endpoint (9.0% versus 11.8%; hazard ratio 0.76, 95% CI 0.62–0.93; *p* = 0.007) and incident diabetes (3.8% versus 7.7%; hazard ratio 0.48, 95% CI 0.33–0.69; *p* < 0.001) [175]. Where canakinumab prevented nothing metabolically, a PPAR-γ agonist prevented diabetes outright in two different populations.

The contrast is instructive, not contradictory, and resolving it sharpens the central claim of this review. The two interventions engage the immunometabolic network at different levels. IL-1β is a terminal, secreted effector with partially redundant substitutes, and neutralising it leaves both the upstream stimulus and the parallel effectors intact. PPAR-γ is an upstream transcriptional node acting in the two cell types that generate the signal in the first place. In the adipocyte, its activation expands subcutaneous storage capacity and redirects lipid away from ectopic depots, so that the glucolipotoxic input described in Section 3 is reduced at source rather than intercepted downstream. In the macrophage, PPAR-γ is required for alternative activation and restrains inflammatory transcriptional output [176]. That the immune arm is load-bearing rather than incidental is shown by the converse experiment: mice lacking PPAR-γ in macrophages alone develop glucose intolerance with hepatic and skeletal muscle insulin resistance on a normal diet, and respond only partially to thiazolidinedione treatment [177]. The antidiabetic effect of the class thus depends in part on an intact macrophage programme.

Two qualifications keep this from being overstated. First, the glycaemic benefit of thiazolidinediones has not been shown to be mediated by their anti-inflammatory action; it is confounded with insulin sensitisation, from which it is inseparable in every trial conducted to date, and the two cannot be disentangled from the available human evidence [38,178]. The class is therefore evidence that intervening upstream of cytokine release can move glycaemia, not evidence that the anti-inflammatory component is what moves it. Second, the same trials that establish the benefit also establish its price: in IRIS, pioglitazone increased weight gain exceeding 4.5 kg (52.2% versus 33.7%), oedema (35.6% versus 24.9%) and fracture requiring surgery or hospitalisation (5.1% versus 3.2%) [175], which is why the class occupies a marginal place in practice despite its mechanistic interest.

The lesson we draw is a constraint on interpretation, not a therapeutic recommendation. The failure of IL-1β neutralisation to modify glycaemia should not be read as evidence that inflammation is metabolically inert, since an agent acting on adipose lipid partitioning and on macrophage transcriptional state does modify it. What appears to determine whether glycaemia moves is the level of the network at which the intervention is applied: upstream, where the inflammatory signal is generated and where substrate flux is set, rather than downstream, at a single secreted effector within a redundant cytokine network.

Hepatokines have emerged as a distinct class of circulating mediators connecting hepatic tissue dysfunction to peripheral insulin resistance, and modulation of specific hepatokine signalling has been proposed as a therapeutic strategy distinct from, but complementary to, the adipose- and gut-directed approaches discussed above, reflecting the broader inter-organ crosstalk framework introduced earlier in this review [179]. Network pharmacology has begun to be applied to traditional multi-component formulations in T2D in order to identify candidate multi-target agents, though without clinical-grade mechanistic validation in human tissue [180]. Overall, the most mechanistically convincing and clinically validated therapeutic strategies in current use—metformin, GLP-1 receptor agonists, and SGLT2 inhibitors—converge on overlapping nodes of the AMPK/mTOR/PI3K-Akt/NF-κB network and the adipose tissue macrophage compartment, reinforcing the centrality of these mechanisms established throughout this review. The primary metabolic actions, candidate immunometabolic effects, molecular targets, and evidence strength of these therapeutic classes are summarised in Table 5.

**Table 5:** Therapeutic targets and immunometabolic intervention points in type 2 diabetes.

Therapeutic Class	Primary Metabolic Action	Immunometabolic Effects	Molecular Targets	Evidence Strength	References
Metformin	Improves glycaemic control and hepatic and whole-body insulin sensitivity	May modulate AMPK-linked inflammatory and mitochondrial pathways	AMPK-linked energy sensing; mitochondrial metabolism; inflammatory mediators	Established glucose-lowering therapy; immunometabolic effects supported mainly by mechanistic and preclinical data.	[148,157,158,159]
GLP-1 receptor agonists	Enhance incretin signalling, weight loss and glucose-dependent insulin secretion	Candidate effects on adipose inflammation, gut metabolic axes and systemic inflammatory tone	GLP-1 receptor signalling; adipose-immune and gut–metabolic mediators	Strong clinical metabolic evidence; immunometabolic mechanisms endpoint-dependent.	[90,155,160,164,171]
SGLT2 inhibitors	Increase urinary glucose excretion and improve cardiometabolic outcomes	Potential modulation of oxidative stress, inflammasome and cytokine signalling and tissue inflammation	SGLT2-linked metabolic shifts; ROS and inflammatory pathways	Strong clinical metabolic and cardiovascular evidence; direct immunometabolic evidence still limited.	[82,166]
Thiazolidinediones/PPAR-γ agonists	Improve adipose insulin sensitivity and lipid partitioning	May shift adipose inflammatory tone and macrophage-adipocyte crosstalk	PPAR-γ; adipocyte differentiation; adipose immune mediators	Strong clinical evidence for metabolic and diabetes-prevention efficacy; immune mediation remains supported mainly by mechanistic and preclinical evidence.	[38,174,175,176,177,178]
Inflammasome- and cytokine-directed approaches	Not primarily glucose-lowering; target inflammatory drivers	May reduce IL-1- and NLRP3-linked inflammatory amplification	NLRP3; caspase-1; IL-1β; IL-18	Mechanistic and emerging early-phase translational evidence.	[16,17,70,71,72]
Microbiome- and gut-barrier interventions	Modify gut-derived signals and metabolic and inflammatory inputs	Potential effects on endotoxaemia, microbial metabolites and systemic inflammation	Gut barrier; LPS and TLR signalling; SCFAs; bile acids	Heterogeneous and context-dependent clinical evidence.	[129,130,131,132,137]

Abbreviations: AMPK, AMP-activated protein kinase; GLP-1, glucagon-like peptide-1; SGLT2, sodium–glucose cotransporter 2; ROS, reactive oxygen species; PPAR-γ, peroxisome proliferator-activated receptor-γ; NLRP3, NOD-, LRR- and pyrin domain-containing protein 3; IL-1β, interleukin-1β; IL-18, interleukin-18; LPS, lipopolysaccharide; TLR, Toll-like receptor; SCFAs, short-chain fatty acids.

## 14. Multi-Omics and Precision Immunometabolism

The mechanistic heterogeneity documented throughout this review—across macrophage activation states, inflammasome activity, mitochondrial function, and islet immune infiltration—has motivated the application of multi-omic and single-cell technologies to resolve immunometabolic subtypes within the clinically heterogeneous syndrome of T2D. Single-cell RNA sequencing has directly revealed immunometabolic alterations in T2D at cellular resolution, identifying specific immune cell subpopulations and transcriptional states associated with disease that are not resolvable by bulk tissue profiling, and providing direct molecular support for the cell-type-specific mechanisms (ATM subpopulations, trained macrophages, islet-resident macrophages) discussed throughout Section 4, Section 6 and Section 8 [18].

High-dimensional immunophenotyping platforms have been developed for metabolic tissue: a 30-colour spectral flow cytometry panel now resolves immune cell composition and macrophage subsets in murine metabolic organs [9]. The resolution such a panel requires indicates what it takes to capture, in a single experiment, the full range of innate and adaptive populations discussed across this review. Such platforms are essential for moving beyond the relatively coarse macrophage polarisation framework historically used to characterise adipose tissue immune remodelling toward a more granular, multi-dimensional characterisation of immune cell state.

Systems-level integration of omics data with computational and artificial-intelligence-based approaches is being explored as a strategy for mapping the pathogenic pathways underlying T2D [181]. The stated goal is to move from descriptive, single-pathway mechanistic models—of the kind synthesised narratively throughout this review—toward integrated, data-driven models that capture the full network of cross-tissue and cross-pathway interactions in disease progression. This computational framing complements the mechanistic synthesis presented in Section 3, Section 4, Section 5, Section 6, Section 7, Section 8, Section 9, Section 10, Section 11, Section 12 and Section 13; it does not replace it, since systems-level models require mechanistically grounded prior hypotheses to be interpretable.

Genetic architecture represents a further, complementary axis of precision stratification that intersects with the immunometabolic mechanisms described throughout this review. Common T2D is highly polygenic. Multi-ancestry meta-analysis across 1.4 million participants identified 318 previously unreported risk loci [144]. Analysis in a still larger multi-ancestry sample then resolved the association signal into mechanistic clusters mapping onto distinct pathophysiological processes: beta-cell dysfunction with and without a proinsulin signature, obesity, lipodystrophy, and liver lipid metabolism. The cluster-specific genetic burden was associated with different patterns of vascular outcome [145]. Monogenic diabetes supplies the complementary limiting case, in which a variant in a single gene defines a discrete aetiological subtype with direct consequences for treatment selection, and which has served as the proof of principle for precision diabetes more generally [182]. Genetic background therefore shapes which of the immunometabolic mechanisms discussed here is likely to predominate in a given individual, and integration of genetic risk stratification with the immune and metabolic profiling approaches described in this section represents a logical, though still largely unrealised, extension of precision immunometabolism [146].

Translational application of these approaches to patient stratification remains at an early stage. Omics-driven discovery continues to identify immune populations and cytokine axes relevant to T2D beyond the macrophage- and lymphocyte-centric framework that has dominated the field—mast cell immunometabolism and IL-17 axis signalling being two recent examples—although their quantitative contribution to human T2D remains to be established [12,183].

Gut microbiota profiling is a further axis of multi-omic characterisation with direct relevance to precision immunometabolism, given the mechanistic centrality of microbiota composition established above. Comprehensive characterisation of the gut microbiota-T2D association, its mechanisms and its translational application has been proposed as a basis for microbiome-informed patient stratification. Robust, validated microbiome-based biomarkers for clinical T2D subtyping have not yet been established [13].

The clinical endpoint of this multi-omic effort is patient stratification by immunometabolic “endotype”: distinguishing, for example, T2D driven predominantly by adipose tissue macrophage-mediated inflammation from T2D driven predominantly by islet-intrinsic inflammatory or mitochondrial mechanisms. The expectation is that such endotypes would respond differentially to the inflammasome-targeted, incretin-based and microbiome-directed therapies discussed in Section 13. At present the stratification is conceptual and not clinically validated: no endotype-stratified trial data demonstrating differential treatment response in T2D have been reported. Data integration across transcriptomic, proteomic, metabolomic and single-cell modalities presents substantial methodological challenges: batch effects, cross-platform normalisation, and the difficulty of establishing causality from cross-sectional snapshots. These constrain how readily such approaches can be translated into the kind of narrative mechanistic synthesis presented here. Quantitative methodology for resolving them in T2D immunometabolic multi-omics has not yet been established.

## 15. Limitations of This Review

Several limitations qualify the conclusions of this review. As a narrative synthesis, it did not apply a systematic search protocol, a formal risk-of-bias assessment, or a quantitative evidence synthesis, and the selection and weighting of evidence therefore reflect an element of interpretive judgement even though the eligibility criteria and the interpretive hierarchy are stated in Section 2. Searching was restricted to PubMed/MEDLINE and Scopus; Embase and Web of Science were not interrogated, and grey literature, preprints and non-English publications were not considered, so that relevant records indexed only in those sources may have been missed. Much of the mechanistic detail integrated here derives from experimental and animal models, in which the temporal and causal relationships between adipocyte stress, macrophage activation, inflammasome signalling, and systemic insulin resistance can be resolved more precisely than in humans; the extent to which the same hierarchy operates in human T2D remains only partly established.

In addition, the immunometabolic framework presented here is deliberately integrative, and the relative quantitative contribution of individual nodes—trained immunity, mitochondrial dysfunction, gut-derived signals, or beta-cell-intrinsic inflammation—to established human disease cannot be ranked with confidence from the current evidence. A third limitation concerns generalisability across the disease itself. As set out in Section 11, the mechanistic chain synthesised here describes the obesity-associated form of T2D, and its applicability to the insulin-deficient, lean and age-related phenotypes that together account for a substantial share of adult-onset diabetes is largely untested; the framework should therefore be read as conditional on phenotype rather than as a general model of the disease. A fourth limitation is specific to the immunometabolic literature on which Section 5 draws. The metabolic states described there were characterised largely in bone marrow-derived murine macrophages exposed to acute, saturating TLR stimulation, a setting that resembles infection more closely than it resembles the chronic, low-intensity lipid stress of adipose tissue in T2D. Human macrophages differ from murine macrophages in surface phenotype, lipid handling, and inflammatory set-point, and the extent to which the succinate–itaconate axis operates as described in human metabolic tissue has not been established. We have presented that framework because it supplies a mechanism the rest of the account requires, but its transferability should be regarded as a hypothesis.

Finally, the therapeutic inferences drawn from the anti-inflammatory properties of existing glucose-lowering agents remain, in several cases, mechanistically plausible without being demonstrated by dedicated immunometabolic endpoints in humans. The trial evidence reviewed in Section 6 and Section 13 should temper this framework more generally: the one strategy derived directly from the mechanism advanced here—sustained IL-1β neutralisation—reduced inflammatory markers and cardiovascular events without preventing incident diabetes. A mechanistic account that is internally coherent, well supported in animal models, and consistent with observational human data can still fail to predict the outcome of the intervention it implies. We regard this as the principal open question raised by our own synthesis, not as a peripheral caveat.

## 16. Conclusions and Future Directions

This review has traced a single, mechanistically continuous chain from nutrient excess to T2D. Glucolipotoxic and danger-associated signals activate innate immune sensors in adipose tissue and other metabolic organs [5,87]. The responding immune cells then reconfigure their own intermediary metabolism, and the metabolites this generates—succinate, and the itaconate that opposes it—themselves determine inflammatory output [59,62]. That output converges on NLRP3 inflammasome assembly, pyroptotic cell death and IL-1β/IL-18 release, which interfere with insulin signalling at a defined molecular lesion: the inhibitory serine phosphorylation of IRS-1 by JNK [30,151,152]. Adipose tissue macrophages and tissue-resident lymphoid populations sustain the resulting state, in part through durable, epigenetically encoded trained immunity [49,51], while mitochondrial dysfunction and impaired mitophagy both follow from it and reinforce it, generating a self-sustaining oxidative-inflammatory loop [7,99]. Beta cells exposed to the same glucolipotoxic and cytokine signals then undergo the immune-cell-mediated and cell-intrinsic dysfunction that converts compensated insulin resistance into overt hyperglycaemia [110,111]. Gut-derived microbial signals feed a further, modifiable systemic input into the whole network [34,124]. This chain describes the obesity-associated form of the disease; its applicability across the other recognised subgroups of T2D is set out, and deliberately left open, in Section 11.

This unifying immunometabolic framework has direct relevance for therapeutic innovation, but its translational claim must be stated with the precision the evidence permits. The mechanistic continuity between established glucose-lowering agents (metformin, GLP-1 receptor agonists, SGLT2 inhibitors) and the inflammatory pathways described throughout this review offers a coherent explanation for metabolic benefits disproportionate to their glycaemic effect alone [16,166]. The converse proposition—that targeting inflammation should by itself improve glycaemia—is the one the human evidence has so far declined to support: IL-1β neutralisation reduced cardiovascular events without preventing incident diabetes [72], and colchicine shows the same vascular-but-not-glycaemic pattern [170]. The rational case for inflammasome-directed agents such as VTX3232 therefore rests on the expectation that earlier intervention, greater pathway selectivity, or tissue-targeted delivery will succeed where systemic cytokine neutralisation in established disease did not [17]—an expectation that is reasonable, but as yet untested.

Several mechanistic gaps emerge as priorities for future investigation. First, the relative quantitative contribution of trained innate immune memory, as opposed to ongoing acute metabolic signalling, to sustained inflammation in established T2D remains incompletely resolved, with direct implications for whether anti-inflammatory interventions need to specifically target epigenetically reprogrammed immune cell populations or whether reducing circulating metabolic substrate would suffice [52,53]. Second, the causal sequence linking adipocyte mitochondrial stress, macrophage activation, and systemic insulin resistance in human (as opposed to experimental) T2D requires further temporal resolution, particularly given evidence that mitochondrial–stress adipocyte-macrophage circuits can sustain metaflammation independent of further nutrient input [31]. Third, the translational gap between mechanistically compelling microbiota-targeted interventions (e.g., Akkermansia muciniphila-derived products, synbiotics) and robust, reproducible clinical efficacy across diverse patient populations remains a significant barrier to adoption of gut-directed therapeutics as primary T2D treatment [130,132]. Fourth, and most consequentially for translation, the trial that would discriminate between the readings of the anakinra–canakinumab divergence set out in Section 6 has not been done. It would combine receptor-level rather than single-ligand blockade, a population with established rather than incipient disease, and beta-cell functional rather than incident-diagnosis endpoints; without it, the question of whether inflammasome-directed therapy can modify glycaemia in any patient group remains open rather than answered in the negative [70,72]. Fifth, the hepatic arm of this immunometabolic network deserves dedicated study: metabolic dysfunction-associated steatotic liver disease (MASLD) shares insulin resistance and chronic low-grade inflammation as core mechanisms with T2D, yet the degree to which hepatic immunometabolic remodelling actively drives, rather than merely accompanies, systemic insulin resistance in patients remains unresolved [184].

Clinically testable hypotheses follow directly from this synthesis. If NLRP3 inflammasome activity is a primary, rather than secondary, driver of the obese hyperglycaemic phenotype, then NLRP3 inhibitors such as VTX3232 should demonstrate glucose-lowering efficacy independent of weight loss in controlled human trials [17]. If islet macrophage-mediated mitochondrial clearance (via pathways such as Reg3g) is protective and not purely degenerative, then promoting this specific macrophage function, in preference to broadly suppressing islet immune cell activity, should preserve beta-cell mass more effectively than non-selective anti-inflammatory approaches [118]. If multi-omic immunometabolic endotyping can reliably stratify patients by dominant pathogenic mechanism (adipose-macrophage-driven versus islet-intrinsic versus gut microbiota-driven), then endotype-matched therapy selection should outperform current uniform treatment algorithms in prospective trials—a hypothesis that, as already noted, current evidence cannot yet test directly, since prospective endotype-matched treatment trials in T2D have not yet been reported. Pending the maturation of such molecular endotyping, accessible clinical phenotyping already identifies patient subgroups in whom hepatic steatosis coincides with subclinical cardiovascular damage, offering a practical—if mechanistically coarse—interim substrate for immunometabolic risk stratification [185].

Finally, immunometabolism provides a unifying mechanistic framework for the obesity-associated form of T2D. It integrates nutrient sensing, innate immune signalling, tissue-specific immune remodelling, mitochondrial biology and beta-cell pathophysiology into a single coherent account, in place of a list of independently studied risk factors. Its extension to the insulin-deficient, lean and genetically distinct subgroups remains a question to be tested, not assumed. The continued application of single-cell and multi-omic technologies to this framework, combined with the ongoing clinical development of inflammasome- and microbiome-directed therapeutics, positions immunometabolism not as a descriptive paradigm but as a candidate basis for the next generation of precision interventions in T2D, conditional on the stratification that those interventions will require [16,18,181].

## Figures and Tables

**Table 1:** Interleukin-1-directed trials with metabolic endpoints: sources of divergence.

	Anakinra (Larsen et al.) [70]	Canakinumab (CANTOS Metabolic Analysis) [71,72]
Agent and molecular target	Recombinant IL-1 receptor antagonist; competitive blockade at IL-1R1—blocks both IL-1α and IL-1β	Monoclonal antibody; neutralisation of IL-1β only
Regimen	100 mg subcutaneously once daily	50, 150 or 300 mg subcutaneously every 3 months
Population	Established T2D (*n* = 70; 34 anakinra, 36 placebo)	Post-infarction, hsCRP ≥ 2 mg/L (*n* = 10,061); pre-diabetes subgroup *n* = 4960 (49.3%)
Glycaemic status at entry	Prevalent hyperglycaemia	Pre-diabetes; primary prevention
Duration	13 weeks	Median follow-up 3.7 years
Endpoint of the analysis	Change in glycated haemoglobin (continuous)	Adjudicated new-onset T2D (dichotomous)
Glycaemic result	HbA1c 0.46 percentage points lower than placebo (*p* = 0.03); C-peptide secretion enhanced (*p* = 0.05); proinsulin-to-insulin ratio reduced (*p* = 0.005)	Incidence 4.2, 4.2, 4.4 and 4.1 per 100 person-years for placebo, 50, 150 and 300 mg (log-rank *p* = 0.84); HR 1.02 (95% CI 0.87–1.19; *p* = 0.82); HbA1c reduced at 6–9 months, not sustained
Insulin sensitivity	Unchanged on hyperinsulinaemic–euglycaemic clamp, as were muscle insulin-regulated gene expression, serum adipokines and body-mass index	Not an endpoint; no consistent long-term effect on fasting plasma glucose
Inflammatory markers	IL-6 reduced (*p* < 0.001); C-reactive protein reduced (*p* = 0.002)	Large reductions in hsCRP and IL-6; target engagement not in doubt
Cardiovascular result	Not assessed	Primary cardiovascular endpoint met
What the study supports	IL-1 signalling contributes causally to beta-cell secretory dysfunction in established disease	IL-1β neutralisation does not prevent conversion to diabetes in pre-diabetes over 3.7 years

Abbreviations: CI, confidence interval; HbA1c, glycated haemoglobin; HR, hazard ratio; hsCRP, high-sensitivity C-reactive protein; IL, interleukin; IL-1R1, interleukin-1 receptor type 1; T2D, type 2 diabetes.

**Table 2:** Immunometabolic mechanisms across tissues in type 2 diabetes.

Mechanism	Main Cells/Tissues	Molecular Mediators	Consequence	References	Evidence Tier
Nutrient excess and metaflammation	Adipocytes; adipose tissue macrophages; liver; skeletal muscle	TNF-α; IL-6; IL-1β; free fatty acids; adipokines; chemokines	Low-grade inflammatory amplification and impaired insulin signalling	[19,20,26,27,28,29]	Animal and in vitro; human observational
Innate immune activation and macrophage remodelling	Monocytes; macrophages; adipose tissue; liver; pancreatic islets	Pattern-recognition receptors; cytokines; chemokines; trained-immunity programmes	Persistent tissue inflammation and metabolic memory-like immune activation	[37,46,52,53,84,85]	Animal and in vitro; some human adipose tissue
Immune cell metabolic reprogramming	Macrophages; monocytes; adipose tissue; pancreatic islets	Glycolytic shift; succinate; HIF-1α; itaconate/ACOD1; mitochondrial ROS	Intracellular metabolism specifies inflammatory versus regulatory phenotype	[58,59,60,61,62]	Animal and in vitro only
NLRP3 inflammasome activation and pyroptosis	Macrophages; adipocytes; endothelial cells; beta cells	NLRP3; caspase-1; IL-1β; IL-18; gasdermin D	Cytokine signalling, cell stress and impaired insulin/beta-cell function	[17,30,64,65,66,80]	Animal and in vitro; human adipose tissue
Mitochondrial dysfunction and oxidative stress	Skeletal muscle; adipose tissue; liver; immune cells; beta cells	ROS; mitochondrial DNA; mitophagy regulators; oxidative phosphorylation defects	Inflammatory pathway activation and reduced metabolic flexibility	[7,32,92,95,99,101]	Animal and in vitro; human observational
Gut barrier dysfunction and metabolic endotoxaemia	Intestinal barrier; microbiota; monocytes/macrophages; liver; adipose tissue	LPS; microbial metabolites; SCFAs; bile acids; TLR signalling	Systemic inflammatory tone and altered glucose homeostasis	[34,67,125,126,128,129]	Animal and in vitro; meta-analysis of randomised trials for dietary fibre
Beta-cell inflammatory stress	Pancreatic beta cells; islet macrophages; immune cells	IL-1β; ER stress; ROS; glucolipotoxic signals; islet cytokines	Impaired insulin secretion, beta-cell dysfunction and beta-cell loss	[70,110,111,112,113,118]	Randomised trial (anakinra); human tissue; animal

Evidence tier follows the hierarchy stated in Section 2: consensus statements and clinical guidelines; randomised controlled trials and meta-analyses; mechanistic studies in human tissue; and experimental or animal studies supporting biological plausibility. Where a row rests on more than one tier, the predominant tier is given first. Abbreviations: TNF-α, tumour necrosis factor-α; IL-6, interleukin-6; IL-1β, interleukin-1β; NLRP3, NOD-, LRR- and pyrin domain-containing protein 3; IL-18, interleukin-18; ROS, reactive oxygen species; DNA, deoxyribonucleic acid; LPS, lipopolysaccharide; SCFAs, short-chain fatty acids; TLR, Toll-like receptor; ER, endoplasmic reticulum; ACOD1, aconitate decarboxylase 1; HIF-1α, hypoxia-inducible factor-1α.

**Table 3:** Recognised sources of heterogeneity in type 2 diabetes and their relation to the immunometabolic chain developed in this review.

Source of Heterogeneity	Defining Features	Relation to the Mechanistic Chain (Section 3, Section 4, Section 5, Section 6, Section 7, Section 8, Section 9 and Section 10)	Hypothesis for Future Testing	References
Obesity-associated T2D (severe insulin-resistant and mild obesity-related subgroups)	Adipose expansion beyond storage capacity, marked insulin resistance, systemic low-grade inflammation.	The chain applies in full, from nutrient excess through inflammasome activation to beta-cell decompensation.	The subgroup in which inflammasome- and macrophage-directed strategies would be expected to have the strongest rationale; not yet tested prospectively.	[5,141]
Severe insulin-deficient diabetes	Early beta-cell failure with low insulin secretion, without marked obesity or insulin resistance.	Entered at the beta-cell node; the upstream adipose and metaflammatory steps are largely bypassed.	Whether islet-directed rather than adipose-directed intervention is required is untested; systemic anti-inflammatory therapy has a correspondingly weaker rationale, but has not been evaluated in this subgroup.	[141,142]
Lean and normal-weight T2D	Body mass index within the normal range, early secretory deficit, clustering in South Asian, sub-Saharan African and Caribbean populations.	Beta-cell immunometabolism (Section 9) may operate with little upstream adipose input.	Whether intra-islet inflammation persists without systemic metaflammation is untested, and determines eligibility for cytokine-directed therapy.	[143]
Mild age-related diabetes	Largest of the data-driven subgroups; later onset, indolent course, modest metabolic derangement.	Neither adipose inflammation nor beta-cell destruction is prominent, and the chain is engaged only weakly.	Whether anti-inflammatory intervention can modify a process in which inflammation is not prominent has not been tested.	[141]
Severe autoimmune diabetes	Autoantibody positivity with early insulin dependence; an adaptive rather than innate immune mechanism.	Outside the scope of this review, which addresses innate immune activation.	A distinct immunological target; conflating it with metaflammation would misdirect therapy.	[141]
Genetically partitioned mechanism	Polygenic burden resolvable into clusters for beta-cell dysfunction, obesity, lipodystrophy and liver lipid handling.	Determines which node of the chain is likely to predominate in a given individual.	Whether genetically defined clusters can support endotype-matched treatment allocation is a hypothesis awaiting prospective evaluation.	[144,145,146]
Ancestry and population differences	Four of the five subgroups recovered in Chinese and United States cohorts in closely similar proportions; the autoimmune cluster was not resolvable in those data.	Supports the reproducibility of the partition beyond European cohorts; does not establish that the weight of each mechanism differs by ancestry.	Whether the mechanisms described here contribute unequally across ancestries requires studies designed to measure it; trial populations require explicit characterisation.	[142,143]

Abbreviations: T2D, type 2 diabetes. The five data-driven subgroups referred to are those of Ahlqvist et al.: severe autoimmune diabetes, severe insulin-deficient diabetes, severe insulin-resistant diabetes, mild obesity-related diabetes, and mild age-related diabetes.

**Table 4:** Core signalling pathways linking nutrient/energy status to inflammatory output.

Pathway	Upstream Triggers	Downstream Effects	Relevance to Insulin Resistance or Beta-Cell Dysfunction	References	Evidence Tier
AMPK	Energy stress; nutrient availability; metformin-associated energetic changes	Metabolic reprogramming, mitochondrial regulation and inflammatory modulation	Links cellular energy state to insulin sensitivity and inflammatory tone	[147,148,154]	Human mechanistic; animal
mTOR	Nutrient excess; amino acids; insulin/leptin signalling; inflammatory stress	Immune-cell metabolic programming, protein synthesis and autophagy regulation	Chronic nutrient signalling can reinforce inflammatory metabolic states	[11,50,58,149]	Animal and in vitro
PI3K/AKT	Insulin signalling; adipokines; inflammatory mediators; AGE-related stress	Glucose uptake, survival signalling and inflammatory crosstalk	Inflammation-associated disruption weakens insulin action	[20,22,42,103,152]	Human mechanistic; animal and in vitro
NF-κB	TLR ligands; cytokines; oxidative stress; saturated fatty acids	Transcription of inflammatory cytokines and chemokines	Central inflammatory transcriptional route connecting immune activation to metabolic dysfunction	[42,49,100,125,150]	Animal and in vitro; human mechanistic
JNK	ER stress; ROS; lipotoxicity; inflammatory cytokines	Stress kinase signalling and interference with insulin pathway components	Stress signalling contributes to insulin resistance and beta-cell stress responses	[21,42,97,151,152]	Animal and in vitro
SIRT1/sirtuins	NAD+ metabolism; caloric/nutrient status; oxidative stress	Mitochondrial and inflammatory regulation through deacetylation programmes	Potential link between metabolic sensing, mitochondrial fitness and inflammatory restraint	[14,153,154]	Animal and in vitro
Succinate–HIF-1α and itaconate	TCA cycle interruption; succinate dehydrogenase; nutrient and TLR signalling	HIF-1α stabilisation, pro-IL-1β transcription and NRF2-dependent antioxidant response	Couples immune cell intermediary metabolism directly to cytokine output	[59,60,61,62]	Animal and in vitro only—LPS-stimulated murine macrophages; not established in human T2D (Section 5 and Section 15)
NLRP3-caspase-1	Mitochondrial ROS; uric acid; lipotoxicity; microbial products; danger signals	IL-1β/IL-18 maturation and pyroptotic inflammatory amplification	Connects danger sensing to cytokine-mediated insulin and islet dysfunction	[30,64,65,66,78]	Animal and in vitro; human adipose tissue

Evidence tier follows the hierarchy stated in Section 2: consensus statements and clinical guidelines; randomised controlled trials and meta-analyses; mechanistic studies in human tissue; and experimental or animal studies supporting biological plausibility. Where a row rests on more than one tier, the predominant tier is given first. Abbreviations: AMPK, AMP-activated protein kinase; mTOR, mechanistic target of rapamycin; PI3K, phosphoinositide 3-kinase; AKT, protein kinase B; AGE, advanced glycation end-product; NF-κB, nuclear factor-κB; TLR, Toll-like receptor; JNK, c-Jun N-terminal kinase; ER, endoplasmic reticulum; ROS, reactive oxygen species; SIRT1, sirtuin 1; NAD+, nicotinamide adenine dinucleotide; NLRP3, NOD-, LRR- and pyrin domain-containing protein 3; IL-1β, interleukin-1β; IL-18, interleukin-18; HIF-1α, hypoxia-inducible factor-1α; NRF2, nuclear factor erythroid 2-related factor 2; TCA, tricarboxylic acid.

## Data Availability

No new data were created or analyzed in this study. Data sharing is not applicable to this article.

## Abbreviations

The following abbreviations are used in this manuscript:

ACOD1	aconitate decarboxylase 1 (IRG1)
AGE	advanced glycation end-product
AMPK	AMP-activated protein kinase
ASC	apoptosis-associated speck-like protein containing a CARD
ATM	adipose tissue macrophage
cGAS	cyclic GMP-AMP synthase
CI	confidence interval
ER	endoplasmic reticulum
FAO	fatty acid oxidation
GLP-1	glucagon-like peptide-1
GSDMD	gasdermin D
HbA1c	glycated haemoglobin
HIF-1α	hypoxia-inducible factor-1α
hsCRP	high-sensitivity C-reactive protein
IL	interleukin
IRS-1	insulin receptor substrate-1
JNK	c-Jun N-terminal kinase
KEAP1	Kelch-like ECH-associated protein 1
LPS	lipopolysaccharide
MASLD	metabolic dysfunction-associated steatotic liver disease
mtDNA	mitochondrial DNA
mTOR	mechanistic target of rapamycin
NF-κB	nuclear factor-κB
NLRP3	NOD-, LRR- and pyrin domain-containing protein 3
NRF2	nuclear factor erythroid 2-related factor 2
OXPHOS	oxidative phosphorylation
PI3K	phosphoinositide 3-kinase
PKC	protein kinase C
PPAR-γ	peroxisome proliferator-activated receptor-γ
ROS	reactive oxygen species
SCFA	short-chain fatty acid
SDH	succinate dehydrogenase
SGLT2	Sodium–glucose cotransporter 2
SIRT1	sirtuin 1
STING	stimulator of interferon genes
T2D	type 2 diabetes
TCA	tricarboxylic acid
TLR	Toll-like receptor
TNF-α	tumour necrosis factor-α
Treg	regulatory T cell
TXNIP	thioredoxin-interacting protein

## Appendix A. Full Search Strings

The searches reproduced below were executed in PubMed/MEDLINE and in Scopus. Searching began in January 2026 and the last search was performed on 20 July 2026. No date, species or article-type restriction was applied at the search stage in PubMed/MEDLINE; eligibility was assessed subsequently against the criteria stated in Section 2. The results of each block were examined by title and abstract in descending order of relevance until further eligible material ceased to be identified, and full texts were obtained for records addressing a mechanism, a clinical endpoint, or an interpretive framework falling within the scope defined in Section 2. The nine blocks were run iteratively rather than as a single combined query, and their results were pooled and de-duplicated.

### Appendix A.1. PubMed/MEDLINE

Block 1—core concept: (“Diabetes Mellitus, Type 2” [Mesh] OR “type 2 diabetes” [tiab] OR T2D[tiab] OR T2DM[tiab]) AND (immunometabolism[tiab] OR immunometabolic[tiab] OR (“Immunity, Innate” [Mesh] AND “Insulin Resistance” [Mesh]))
Block 2—metaflammation and innate immune activation: (“Diabetes Mellitus, Type 2” [Mesh] OR “Insulin Resistance” [Mesh] OR “Obesity” [Mesh]) AND (metaflammation[tiab] OR “meta-inflammation” [tiab] OR “low-grade inflammation” [tiab] OR “Inflammation” [Mesh]) AND (“Macrophages” [Mesh] OR macrophage* [tiab] OR “Toll-Like Receptors” [Mesh])
Block 3—inflammasome, pyroptosis and cytokine signalling: (NLRP3[tiab] OR inflammasome* [tiab] OR “Pyroptosis” [Mesh] OR gasdermin* [tiab] OR “Interleukin-1beta” [Mesh]) AND (“Diabetes Mellitus, Type 2” [Mesh] OR “Insulin Resistance” [Mesh] OR “Insulin-Secreting Cells” [Mesh] OR “Adipose Tissue” [Mesh])
Block 4—metabolic reprogramming of immune cells: (macrophage* [tiab] OR “Myeloid Cells” [Mesh]) AND (“Glycolysis” [Mesh] OR glycolysis[tiab] OR succinate* [tiab] OR itaconate* [tiab] OR “Citric Acid Cycle” [Mesh] OR “Hypoxia-Inducible Factor 1, alpha Subunit” [Mesh]) AND (inflammat* [tiab] OR polaris* [tiab] OR polariz* [tiab])
Block 5—mitochondria, reactive oxygen species and mitophagy: (“Mitochondria” [Mesh] OR mitochondri* [tiab] OR “Mitophagy” [Mesh] OR mitophagy[tiab] OR “Reactive Oxygen Species” [Mesh] OR “DNA, Mitochondrial” [Mesh] OR cGAS[tiab] OR STING[tiab]) AND (“Diabetes Mellitus, Type 2” [Mesh] OR “Insulin Resistance” [Mesh] OR “Adipose Tissue” [Mesh] OR “Insulin-Secreting Cells” [Mesh])
Block 6—beta-cell immunometabolism: (“Insulin-Secreting Cells” [Mesh] OR “beta cell*” [tiab] OR “Islets of Langerhans” [Mesh]) AND (inflammat* [tiab] OR “Cytokines” [Mesh] OR macrophage* [tiab] OR glucolipotoxic* [tiab] OR lipotoxic* [tiab])
Block 7—gut-derived signals: (“Gastrointestinal Microbiome” [Mesh] OR microbiot* [tiab] OR “Lipopolysaccharides” [Mesh] OR endotoxaemia[tiab] OR endotoxemia[tiab] OR “Fatty Acids, Volatile” [Mesh] OR “short-chain fatty acid*” [tiab] OR “Bile Acids and Salts” [Mesh]) AND (“Diabetes Mellitus, Type 2” [Mesh] OR “Insulin Resistance” [Mesh] OR “Obesity” [Mesh])
Block 8—therapeutic classes: (“Metformin” [Mesh] OR “Glucagon-Like Peptide-1 Receptor” [Mesh] OR “glucagon-like peptide 1 receptor agonist*” [tiab] OR “Sodium-Glucose Transporter 2 Inhibitors” [Mesh] OR canakinumab[tiab] OR anakinra[tiab] OR colchicine[tiab] OR dapansutrile[tiab] OR “NLRP3 inhibitor*” [tiab] OR “Thiazolidinediones” [Mesh]) AND (inflammat* [tiab] OR immunomodulat* [tiab] OR macrophage* [tiab] OR inflammasome* [tiab])
Block 9—heterogeneity and precision medicine: (“Diabetes Mellitus, Type 2” [Mesh]) AND (heterogene* [tiab] OR “cluster analysis” [tiab] OR endotype* [tiab] OR subtype* [tiab] OR “Precision Medicine” [Mesh] OR “Multiomics” [Mesh] OR “Single-Cell Analysis” [Mesh] OR “Genome-Wide Association Study” [Mesh])

### Appendix A.2. Scopus

The same nine concept blocks were translated into Scopus syntax using the TITLE-ABS-KEY field code. In Scopus, unlike PubMed/MEDLINE, language and document-type limits were applied at the search stage, as shown in each string. The nine strings executed were as follows.

Block 1—core concept: TITLE-ABS-KEY ((“type 2 diabetes” OR “T2D” OR “T2DM” OR “insulin resistance”) AND (“immunometabolism” OR “immunometabolic” OR (“innate immunity” AND “insulin resistance”))) AND (LIMIT-TO (LANGUAGE, “English”)) AND (LIMIT-TO (DOCTYPE, “ar”) OR LIMIT-TO (DOCTYPE, “re”))
Block 2—metaflammation and innate immune activation: TITLE-ABS-KEY ((“type 2 diabetes” OR “T2D” OR “T2DM” OR “insulin resistance”) AND ((“metaflammation” OR “meta-inflammation” OR “low-grade inflammation”) AND (“macrophage*” OR “toll-like receptor*”))) AND (LIMIT-TO (LANGUAGE, “English”)) AND (LIMIT-TO (DOCTYPE, “ar”) OR LIMIT-TO (DOCTYPE, “re”))
Block 3—inflammasome, pyroptosis and cytokine signalling: TITLE-ABS-KEY ((“type 2 diabetes” OR “T2D” OR “T2DM” OR “insulin resistance”) AND (“NLRP3” OR “inflammasome*” OR “pyroptosis” OR “gasdermin*” OR “interleukin-1beta”)) AND (LIMIT-TO (LANGUAGE, “English”)) AND (LIMIT-TO (DOCTYPE, “ar”) OR LIMIT-TO (DOCTYPE, “re”))
Block 4—metabolic reprogramming of immune cells: TITLE-ABS-KEY ((“type 2 diabetes” OR “T2D” OR “T2DM” OR “insulin resistance”) AND ((“glycolysis” OR “succinate” OR “itaconate” OR “tricarboxylic acid cycle” OR “HIF-1alpha”) AND (“macrophage*”))) AND (LIMIT-TO (LANGUAGE, “English”)) AND (LIMIT-TO (DOCTYPE, “ar”) OR LIMIT-TO (DOCTYPE, “re”))
Block 5—mitochondria, reactive oxygen species and mitophagy: TITLE-ABS-KEY ((“type 2 diabetes” OR “T2D” OR “T2DM” OR “insulin resistance”) AND (“mitochondri*” OR “mitophagy” OR “reactive oxygen species” OR “mitochondrial DNA” OR “cGAS” OR “STING”)) AND (LIMIT-TO (LANGUAGE, “English”)) AND (LIMIT-TO (DOCTYPE, “ar”) OR LIMIT-TO (DOCTYPE, “re”))
Block 6—beta-cell immunometabolism: TITLE-ABS-KEY ((“type 2 diabetes” OR “T2D” OR “T2DM” OR “insulin resistance”) AND ((“beta cell*” OR “islet*”) AND (“inflammat*” OR “cytokine*” OR “macrophage*” OR “glucolipotoxicity”))) AND (LIMIT-TO (LANGUAGE, “English”)) AND (LIMIT-TO (DOCTYPE, “ar”) OR LIMIT-TO (DOCTYPE, “re”))
Block 7—gut-derived signals: TITLE-ABS-KEY ((“type 2 diabetes” OR “T2D” OR “T2DM” OR “insulin resistance”) AND (“microbiot*” OR “lipopolysaccharide” OR “endotoxaemia” OR “endotoxemia” OR “short-chain fatty acid*” OR “bile acid*”)) AND (LIMIT-TO (LANGUAGE, “English”)) AND (LIMIT-TO (DOCTYPE, “ar”) OR LIMIT-TO (DOCTYPE, “re”))
Block 8—therapeutic classes: TITLE-ABS-KEY ((“type 2 diabetes” OR “T2D” OR “T2DM” OR “insulin resistance”) AND ((“metformin” OR “GLP-1 receptor agonist*” OR “SGLT2 inhibitor*” OR “canakinumab” OR “anakinra” OR “colchicine” OR “dapansutrile” OR “NLRP3 inhibitor*” OR “thiazolidinedione*”) AND (“inflammat*” OR “immunomodulat*” OR “inflammasome*”))) AND (LIMIT-TO (LANGUAGE, “English”)) AND (LIMIT-TO (DOCTYPE, “ar”) OR LIMIT-TO (DOCTYPE, “re”))
Block 9—heterogeneity and precision medicine: TITLE-ABS-KEY ((“type 2 diabetes” OR “T2D” OR “T2DM” OR “insulin resistance”) AND (“heterogeneity” OR “cluster analysis” OR “endotype*” OR “subtype*” OR “precision medicine” OR “multi-omics” OR “single-cell” OR “genome-wide association”)) AND (LIMIT-TO (LANGUAGE, “English”)) AND (LIMIT-TO (DOCTYPE, “ar”) OR LIMIT-TO (DOCTYPE, “re”))

Reference lists of the records retained from these searches, and of the major reviews identified by them, were screened manually; records identified in this way are cited throughout on the same eligibility criteria as those retrieved by database searching.
